# High Ano1 expression as key driver of resistance to radiation and cisplatin in HPV-negative head and neck squamous cell carcinoma

**DOI:** 10.1038/s41598-025-85214-9

**Published:** 2025-01-10

**Authors:** Solenne Bourdier, Anne-Sophie Fisch, Keziban Merve Alp, Ridhima Das, Philipp Mertins, Ingeborg Tinhofer

**Affiliations:** 1 Department of Radiooncology and Radiotherapy, Charité – Universitätsmedizin Berlin, Corporate Member of Freie Universität Berlin and Humboldt-Universität zu Berlin, Charitéplatz 1, 10117 Berlin, Germany; 2 Max Delbrück Center for Molecular Medicine, Robert-Rössle-Str. 10, 13092 Berlin, Germany; 3 German Cancer Research Center (DKFZ), German Cancer Consortium (DKTK) Partner Site Berlin, 69120 Heidelberg, Germany

**Keywords:** HNSCC, 11q13 amplification, Anoctamin-1, TMEM16A, Radiosensitivity, Mass spectrometry, Radiotherapy, Cancer therapeutic resistance, Cancer genomics, Mechanisms of disease, Predictive markers

## Abstract

Human papilloma virus-negative head and neck squamous cell carcinoma (HNSCC) frequently harbors 11q13 amplifications. Among the oncogenes at this locus, *CCND1* and *ANO1* are linked to poor prognosis; however, their individual roles in treatment resistance remain unclear. The impact of Cyclin D1 and Ano1 overexpression on survival was analyzed using the TCGA HNSCC dataset and a Charité cohort treated with cisplatin (CDDP)-based radiochemotherapy. High Ano1 expression was primarily associated with poor overall survival in both datasets. The effects of *CCND1* and *ANO1* knockdown (KD) on radio- and drug sensitivity, along with changes in global protein expression, cell viability, growth, and DNA repair, were studied in an 11q13-amplified HNSCC cell line model of primary cisplatin resistance. Unique pathway alterations– VEGF in *CCND1* KD and the Rho GTPase cycle in *ANO1* KD– were observed, along with shared changes like DNA damage and cell cycle dysregulation. Silencing *CCND1* or *ANO1* increased CDDP sensitivity, while only *ANO1* silencing increased radiosensitivity. Copanlisib and afatinib were identified as promising candidates for combination therapy of 11q13-amplified HNSCC tumors. We demonstrated a predominant role for Ano1 in treatment resistance in Cyclin D1^high^Ano1^high^ HNSCC tumors and identified novel potential treatment combinations for this high-risk patient group.

**Supplementary Information:**

The online version contains supplementary material available at 10.1038/s41598-025-85214-9.

## Introduction

With approximately 900,000 cases per year, Head and Neck Squamous Cell Carcinoma (HNSCC) ranks as the 7th most frequent cancer globally^1^. It originates from the epithelial mucosa of the larynx, hypopharynx, oropharynx, nasopharynx, and oral cavity. The incidence is primarily linked to alcohol and tobacco consumption, as well as human papillomavirus (HPV) infection. First-line treatment for HNSCC often includes surgery and radiotherapy, with or without the addition of platinum-based chemotherapy^2^. Despite significant improvements in treatment, the 5-year overall survival rate remains below 50% for the HPV-negative subgroup, compared to 80% for the HPV-positive subgroup^3,4^. The high recurrence rate after radiochemotherapy (RCTx) in HPV-negative patients underscores the need for better therapeutic strategies for this unfavorable group. High interpatient heterogeneity in the molecular characteristics of HNSCC tumors complicates further treatment optimization.

As previously reviewed, amplification of the 11q13 locus is predictive for poor outcome in patients with HNSCC^5^. It occurs in about 30% of HPV-negative HNSCC patients (cBioPortal, HNSCC TCGA PanCancer Atlas database). The 11q13 locus encompasses several well-characterized genes, including: *CCND1*, *ORAOV1*, *FGF19*, *FGF4*, *FGF3*, *ANO1*, *FADD*, *PPFIA1*, and *CTTN*, all co-amplified in HNSCC tumors^5^. These genes are involved in crucial signaling pathways for tumor cell survival, proliferation, apoptosis, and cell migration^5,6^, making them potential therapeutic targets for treatment optimization.

Cyclin D1, encoded by *CCND1*, is a critical regulator of the cell cycle, contributing to uncontrolled cellular proliferation in cancer. Cyclin D1 is closely associated with the CDK4/6 complex, which phosphorylates the retinoblastoma protein (Rb). When phosphorylated, pRb detaches from the elongation factor E2F, allowing cell cycle progression and proliferation^7^. Although Cyclin D1 overexpression cannot be targeted directly, its deregulated functions in 11q13 amplified cases might be indirectly targeted by inhibiting the CDK4/6 complex.

Another significant gene at the 11q13 locus is *ANO1* (synonym: TMEM16A), which encodes Anoctamin-1 (referred to herein as Ano1), a transmembrane calcium-activated chloride channel involved in essential physiological functions of various tissues including salivary glands^8^ and airway epithelium^9^. One key feature of Ano1 is its ability to form a tight complex with the epidermal growth factor receptor (EGFR), thereby stabilizing EGFR expression^10^. Since EGFR is overexpressed in 90% of HNSCC cases^11^, and its overexpression is associated with poor prognosis^12,13^, targeting Ano1 might represent a preferentially attractive therapeutic strategy for 11q13 amplified HNSCC^14,15^.

While targeting oncogenic drivers such as Cyclin D1 and Ano1 could be an attractive therapeutic strategy to improve outcomes in HPV-negative HNSCC, the high co-expression of these genes obscures their individual impact on radio and chemo-resistance. Analysis of patient outcome according to gene expression in the TCGA HNSCC dataset as well as a Charité patient cohort indicated a major role of Ano1 overexpression in poor prognosis. Using a previously established syngenic 11q13 amplified HNSCC cell line model of primary cisplatin (CDDP) resistance, we thus investigated in detail the role of *CCND1* and *ANO1* amplification in treatment resistance. Gene silencing by shRNA was combined with proteome analysis by mass spectrometry and functional phenotype analysis, including assessment of sensitivity to irradiation and CDDP.

## Materials and methods

### Cell lines

Four HPV-ve cell lines were selected according to their 11q13 amplification status: four single-cell derived subclones from the hypopharyngeal tumor cell line FaDu (ATCC^®^HTB–43™, purchased from ATCC, Manassas, VA, USA) displaying sensitivity (clone 46, c46; clone 54, c54) or primary resistance to CDDP (clone 5, c5; clone 78, c78) which had been established and comprehensively characterized in a previous study^16^ UM-SCC-22B, UT-SCC-9 and UT-SCC-15 (kindly provided by Prof. Dr. Kathrin Scheckenbach, University of Düsseldorf). Cells were maintained at 37 °C supplied with 5% CO_2_ in a humidified atmosphere. Media composition with references of the reagents are described in Supplementary Materials and Methods: Media composition. All cells were regularly tested by PCR for mycoplasma contamination (Applied Biological Materials abm, Richmond, Canada). Drugs used for drug testing included cisplatin (NeoCorp, Hexal, Holzkirchen, Germany), palbociclib, afatinib, copanlisib (all from Selleckchem, Cologne, Germany), and MG-132 (Hycultec, Beutelsbach, Germany).

### Copy number analysis by droplet digital PCR (ddPCR)

Genomic DNA was extracted based on the manufacturer’s instructions (Qiagen, Hilden, Germany). The ddPCR data were processed using QuantaSoft™ software (version 1.7.4, BioRad, Hercules, CA, USA). All experiments were performed in three independent replicates. Sequences of the primers and probes, PCR parameters and reagents are described in Supplementary Materials and Methods: Droplet Digital PCR.

### Gene expression analysis by RT-qPCR

RNA was extracted from non-confluent cultured cells and was quantified on a LightCycler 480 II system (Roche, Basel, Switzerland). Sequences of the primers and probes, PCR parameters and reagents are described in Supplementary Materials and Methods: RT-qPCR.

### Protein detection by western blot

Proteins were quantified as described in Supplementary Materials and Methods: Western Blot, as well as the antibodies list.

### Construction of shRNA plasmids

shRNA targeting the genes of interest were cloned into a LeGo vector containing a green fluorescence protein (GFP) cassette (LeGo-Lentiviral Gene Ontology Vectors, Kristoffer Riecken, Hamburg, Germany). shRNA sequences and description of the lentivirus transduction are provided in Supplementary Materials and Methods: shRNA sequences, transfection of lentivirus and cell transduction and generation of stable cell lines.

###  Microscopy imaging

Bright field microscopy using a 10x objective lens was used in order to measure organoid diameters or monitoring cell growth under different conditions, and fluorescence microscopy for assessing transduction efficiency based on the cell’s GFP signal (Leica DFC3000 G system, Wetzlar, Germany).

### Cell cycle analysis

Cells were detached with Trypsin-EDTA (Merck, Darmstadt, Germany) washed in PBS and fixed in 70% (vol/vol) ethanol overnight at -20 °C, washed twice with PBS and incubated for 30 min with 50 µg/ml RNase (Thermo Fisher Scientific, Waltham, MA, USA). DNA was stained with 10 µM DRAQ5 (Miltenyi Biotec, Bergisch Gladbach, Germany) and cells were analyzed with a flow cytometer (BD Biosciences FACSCanto II, Franklin Lakes, NJ, USA). Cell cycle distribution was analyzed using the software FlowJo (version 7.6.5, BD Biosciences).

### Drug testing of 2D and 3D models

For drug testing in 2D cultures, 3 × 10^3^ cells were seeded per well in a 96-well plate and treated 24 h after seeding with different concentrations of inhibitor, drug or solvent control. After 48–72 h, metabolic activity of cells was measured using the MTT assay. Briefly, 3-(4,5-dimethylthiazol-2-yl)-2,5-diphenyltetrazolium bromide reagent (MTT, Roth, Karlsruhe, Germany) was added to the cells. After 2 h of incubation, formazan was dissolved with DMSO and absorbance was measured at 540 nm by plate reader (PerkinElmer Victor Nivo™ Control Software: 2.5.4., Waltham, MA, USA). For 3D models, 1 × 10^3^ cells per 5 µL ice-cold Matrigel drop (Corning, Corning, NY, USA) were seeded in 96-well plate and let grow for 96 h until organoids reached ~ 35 μm diameter. Drugs were added and incubated for 48 to 72 h. Cell viability was determined using the CellTiter-Glo Luminescent assay (Promega, Madison, WI, USA) according to the manufacturer’s instructions, adapted for 3D cultures by increasing the incubation time to 30 min. Luminescence was detected at 700 nm with Victor Nivo plate reader. Non-linear regression curves were plotted to determine the IC_50_ value from three independent experiments (six technical replicates each) using GraphPad Prism 8 (version 8.4.3, Boston, MA, USA). Synergy scores were determined with SynergyFinder Plus software, where CI < 1 represented synergistic effect.

### Clonogenic survival assays

Before irradiation, cells were seeded at a density of 1 × 10^3^ cells per well in a 6-well plate, or 250 cells per well in a 12-well plate. After 24 h post seeding, cells were subjected to X-ray irradiation using single doses of up to 6 Gy delivered by YXLON Maxishot (YXLON International GmbH, Hamburg, Germany) at 0.89 Gy/min. Cells were cultured in medium for another 14 days until the medium was removed. Cells were washed with PBS, fixed with 70% ethanol and stained with 10% Giemsa solution (Merck). Colonies containing 50 cells or more were counted. Plating efficiency (PE) was calculated as the number of colonies divided by the number of seeded cells. Survival fraction (SF) were calculated as the PE of the treated cells divided by the PE of the control. Clonogenic survival curves were fitted by the linear-quadratic model, and D0 (or D37) representing the dose in Gy required to reduce the SF to 37% as well as the area under the curve (AUC) were calculated by GraphPad Prism for each cell line. Experiments were performed in three biological replicates and three technical replicates per condition.

### Cell growth kinetic by live cell imaging

Cells were seeded at a density of 2 × 10^5^ cells per well in a 6-well plate. Following their adherence, cells were imaged every 3 h for 5 days with a 10x objective in an Incucyte SX5 G/R Optical Module Live-Cell Analysis System (Sartorius, Göttingen, Germany). The confluence of cell monolayers was measured with the Incucyte software (version 2022 A).

### VEGF-ELISA quantification

Cells were treated with copanlisib at the indicated concentrations. After 24 h and 48 h, 1 mL supernatant was removed, centrifuged at 1500 rpm for 5 min to remove debris and kept at -20 °C for VEGF level quantification. Samples were analyzed based on the manufacturer instructions VEGF Human VEGF Quantikine ELISA (R&D Systems, Minneapolis, MN, USA). The amount of VEGF was quantified using a plate reader (BioTek Synergy HTX, Agilent Technologies, Santa Clara, CA, USA) set at 450 nm and applying a wavelength correction set at 540 nm substracted from the readings at 450 nm.

### γH2AX foci analysis

For microscope characterization of γH2AX level, 2 × 10^5^ cells were seeded on a sterile 13 mm round glass cover slip placed at the bottom of a 24-well plate. After 24 h cells were irradiated at 4 Gy and let grown 24 h before fixation with 4% paraformaldehyde, done directly on the cover slip. Cells were permeabilized 30 min at 4 °C in PBS supplemented with 1% BSA and 0.1% Triton X-100. Alexa Fluor^®^ 647 anti-H2AX Phospho (Ser139) antibody clone 2F3 (BioLegend, San Diego, CA, USA) was added at a 1:200 dilution for 2 h incubation. After washing with PBS, cells were stained with Hoechst 33342 (dilution 1:500, Thermo Fisher Scientific). For imaging, cover slips were flipped on SuperFrost Plus GOLD (Menzel-Gläser, Braunschweig, Germany) covered with mounting medium (ROTI^®^Mount FluorCare, Roth). Cell images were acquired with a Yokogawa CQ1 confocal imaging microscope, equipped with a 40x objective (Yokogawa, Musashino, Japan). Nuclei and foci were automatically counted using NIS Elements software (version 5.30.02, Nikon, Minato, Japan). Pixels were converted to µm^2^ to calculate nuclear area. Small nuclei in the lower 25% quantile and outliers were excluded. The number of foci per nuclear area was determined from at least 300 nuclei.

### Mass spectrometry analysis

Frozen cell pellets were lysed in urea buffer (8 M urea, 75 mM NaCl, 50 mM Tris, 1 mM EDTA, protease inhibitors). After vortexing, samples were incubated on ice for 30 min. The supernatant was collected following centrifugation and protein concentration was measured via BCA assay. For mass spectrometry, 50 µg of protein per sample was used. Samples were reduced with 5 mM DTT for 1 h at 37 °C, then alkylated with 10 mM IAA for 45 min at 25 °C in the dark. Urea concentration was reduced to 2 M with 50 mM Tris-HCl before Lys-C digestion (1:50 enzyme to substrate) for 2 h, followed by overnight trypsin digestion (1:50 enzyme to substrate) at 25 °C. The reaction was stopped with 1% formic acid (FA). Peptides were desalted using StageTips with C18 material and reconstituted in 3% acetonitrile (ACN)/0.1% FA to 0.5 µg/µL. 1 µg of peptides was analyzed by LC-MS/MS using an Orbitrap Exploris 480 coupled to an Easy nLC 1200 system. Peptides were separated over a 110-min gradient on a 20 cm reverse-phase column packed with 1.9 μm beads. The mass spectrometer operated in data-independent acquisition mode. Data were processed with Spectronaut software using directDIA workflow, a widely accepted method for label-free data-independent acquisition (DIA), in default settings, matched against the human UniProt FASTA and contaminants databases. The protein report was used for further analysis. Normalization was achieved through median centering, where the median protein intensity in each sample was calculated and adjusted to ensure consistent median values across samples. Approximately 7,000–10,000 proteins were identified per run in the cell line experiments.

Label Free Quantification (LFQ) intensity obtained from three independent replicates was used to calculate the fold-changes of each protein. Unpaired t-test was used to determine significant changes between knockdown and control cells. q-values were derived from *P*-values by adjusting for the False Discovery Rate. A cutoff of 0.01 was applied for q-values and proteins presenting a fold change below 0.5 or above 2 were selected for gene enrichment analysis.

### Statistical analysis

Statistical analysis was done using the SPSS (version 29.0.0.0 (241)) and GraphPad Prism 8 (version 8.4.3) softwares. Two group-comparisons were performed using unpaired two-tailed Student’s t tests. Results were considered statistically significant when *p*-values were < 0.05. Significance levels were indicated as follows: * (*P* < 0.05), ** (*P* < 0.01), *** (*P* < 0.001), and **** (*P* < 0.0001).

##  Results

### High expression of Ano1 in the absence of Cyclin D1 overexpression drives poor outcome in HPV-ve HNSCC patients.

**Fig. 1:**
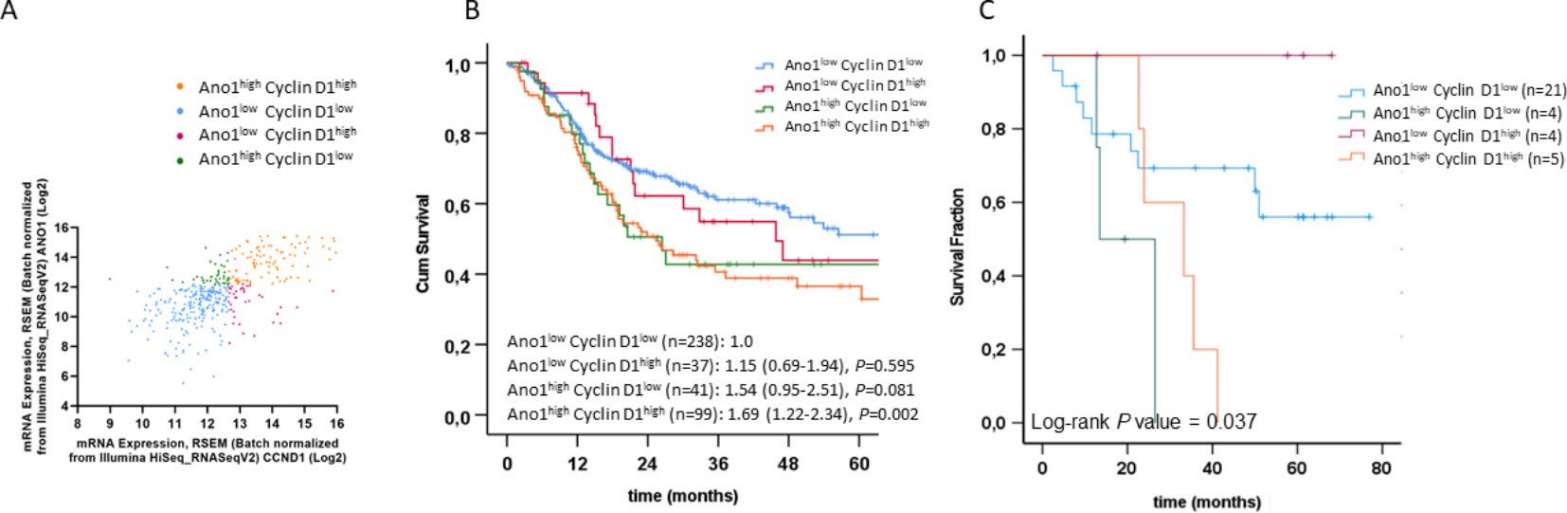
High Ano1 expression in the absence of high cyclin D1 is associated with poor prognosis in HPV-negative HNSCC. (**A**,** B**) Patients (HPV- negative cases from the TCGA HNSCC PanCancer Atlas dataset; *n* = 415) were classified in four groups based on the expression levels of Ano1 and Cyclin D1. (**A**) Correlation between Cyclin D1 and Ano1 mRNA expression levels; (**B**) Kaplan Meier curves and Cox regression hazard ratios (+/- 95% confidence intervals) for overall survival according to Ano1 and Cyclin D1 expression levels; (**C**) Kaplan Meier curves of overall survival for locally advanced HNSCC patients treated with CDDP-based RCTx (Charité cohort, *n* = 34) according to Ano1 and Cyclin D1 expression levels are shown.

The HPV-ve cohort from the TCGA HNSCC PanCancer dataset, including 415 patients, was used for assessing the potential distinct roles of Cyclin D1 and Ano1 overexpression in disease outcome. As expected, the correlation of mRNA levels showed coexpression between Cyclin D1 and Ano1 (Pearson coefficient: 0.64, *P* = 2.4e^-58^) (Fig. 1A). However, approximately 9% of cases exhibited high expression of Ano1 but low Cyclin D1 expression, and vice versa. Using the mRNA z-score, which represents the standard deviation from the mean, with mRNA expression levels log-transformed and normalized across all samples (log RNA Seq V2 RSEM), patients were stratified into four groups based on their Cyclin D1 and Ano1 expression levels. A threshold level of > 1.5 was applied to evaluate the effects of Cyclin D1 and Ano1 in the highest expressing groups. Kaplan-Meier analysis revealed poor overall survival (OS) in the group of patients with high Ano1 expression even in the absence of high Cyclin D1 expression levels, with hazard ratios only slightly higher in the patient group with high expression of both genes. In contrast, no difference in OS was observed between Ano1^low^-Cyclin D1^high^ and the Ano1^low^-Cyclin D1^low^ groups (Fig. 1B). The TCGA HNSCC dataset comprises patients treated with standard treatment modalities including surgery and/or radiotherapy alone or in combination with chemotherapy. We therefore also performed an exploratory analysis in the subset of TCGA HNSCC patients receiving platinum (DDP)-based RCTx (*n* = 101). As in the whole dataset, high Ano1 expression in the absence of Cyclin D1 overexpression was associated with worse outcome (Supplementary Figure S1). Since numbers in the strata were small and comprised patients treated with definitive or adjuvant RCTx, we repeated this analysis in a Charité patient cohort (*n* = 34) of locally advanced HNSCC uniformly treated with definitive CDDP-based RCTx, and could confirm the results of the TCGA HNSCC dataset analysis (Fig. 1C). Based on these results, we hypothesized that Ano1 expression has a stronger impact on tumor cell sensitivity to radiation and CDDP, an assumption we tested in HNSCC cell line models.

###  Stable knockdown of CCND1 and ANO1 by shRNA

We next focused on mechanistically analyzing the roles of Ano1 and Cyclin D1 in radio- and CDDP-resistance. We primarily utilized the FaDu cell line model, which harbors the 11q13 amplification, along with CDDP-resistant (c5 and c78) and CDDP-sensitive (c46 and c54) subclones previously generated in our laboratory^16^. For comparative analysis, we included an additional cell line with *CCND1*/*ANO1* amplifications (UM-SCC-22B), as well as non-amplified cell lines (UT-SCC-9 and UT-SCC-15). The respective genotypes were confirmed in all models (Supplementary Figure S2 A-D). The CDDP-resistant FaDu subclone c5 was subjected to *CCND1* and *ANO1* knockdown (KD) or scramble RNA (control) shRNA lentivirus-mediated transduction. The high efficacy of the KD was confirmed by qPCR analysis (Supplementary Figure S2 E-F).

### Cyclin D1 and Ano1 regulate DNA damage repair as well as cell cycle pathways

**Fig. 2:**
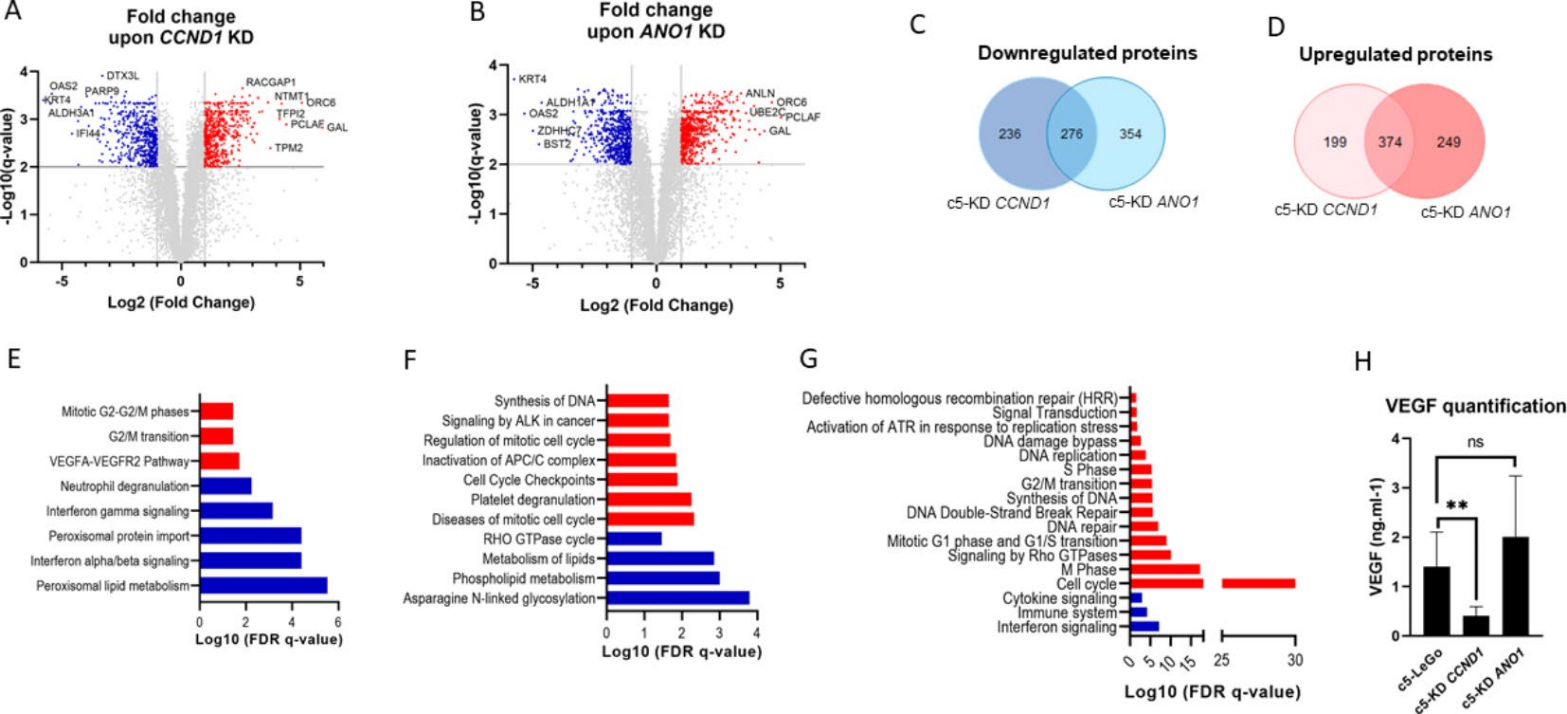
Proteome analysis revealed common and unique protein expression changes upon*CCND1*or*ANO1*silencing. (**A, B**) Volcano plots of fold changes upon *CCND1*(**A**) or *ANO1*(**B**) KD compared to LeGo, with significant upregulated proteins in red and downregulated proteins in blue; (**C**,** D**) Venn diagrams illustrating the number of common and unique proteins that were down- (**C**) or up-regulated (**D**) upon KD; (**E-G**) Pathway enrichment analysis referred to the Reactome dataset based on uniquely down- (blue) and upregulated proteins (red) upon *CCND1* KD (**E**) and *ANO1* KD (**F**), or common for both KD (**G**). Only the significant pathways (FDR < 0.05) are shown; (**H**) VEGF detection by ELISA upon *CCND1* KD. Bars represent ± SEM from at least three independent experiments.

To evaluate the molecular changes resulting from *CCND1* and *ANO1* silencing, we conducted a proteomic analysis using mass spectrometry (Fig. 2). Volcano plots revealed that 514 proteins were significantly downregulated, and 573 proteins upregulated following *CCND1* KD (Fig. 2A), while 630 proteins were downregulated and 623 upregulated upon *ANO1* KD (Fig. 2B). Venn diagrams illustrate the numbers of unique and common proteins affected by *CCND1* or *ANO1* KD (Fig. 2C-D), which were subsequently used for gene enrichment analysis. *CCND1* KD significantly impacted the mitotic G2/M phase, interferon signaling, and vascular endothelial growth factor (VEGF) signaling pathways (Fig. 2E), whereas Ano1 depletion altered proteins associated with DNA synthesis, the Rho GTPase cycle, and lipid metabolism (Fig. 2F). Common gene enrichment features found in both knockdowns were linked to DNA damage repair and synthesis, cell cycle transition and cytokine signaling (Fig. 2G). Given that high expression of VEGF is associated with drug resistance^17^, we validated our mass spectrometry findings using an ELISA assay. We confirmed that *CCND1* KD significantly reduced VEGF levels compared to the LeGo control, while VEGF levels remained unaffected by *ANO1* KD (Fig. 2H). Although VEGF protein levels were downregulated according to the ELISA assay, downstream effector proteins involved in the VEGF signaling pathway were upregulated, likely reflecting a compensatory mechanism following VEGF downregulation. The upregulated proteins included BAIAP2 (2-fold), AXL (2.16-fold), MAPKAPK3 (2-fold), CAV1 (2.79-fold), ELMO2 (2.22-fold), PXN (2.71-fold) and VAV2 (2.21-fold). In summary, these results demonstrate that *CCND1* and *ANO1* silencing leads to both common and unique protein expression changes in HNSCC cells.

###  Both cyclin D1 and Ano1 expression interfere with cell viability and growth

**Fig. 3:**
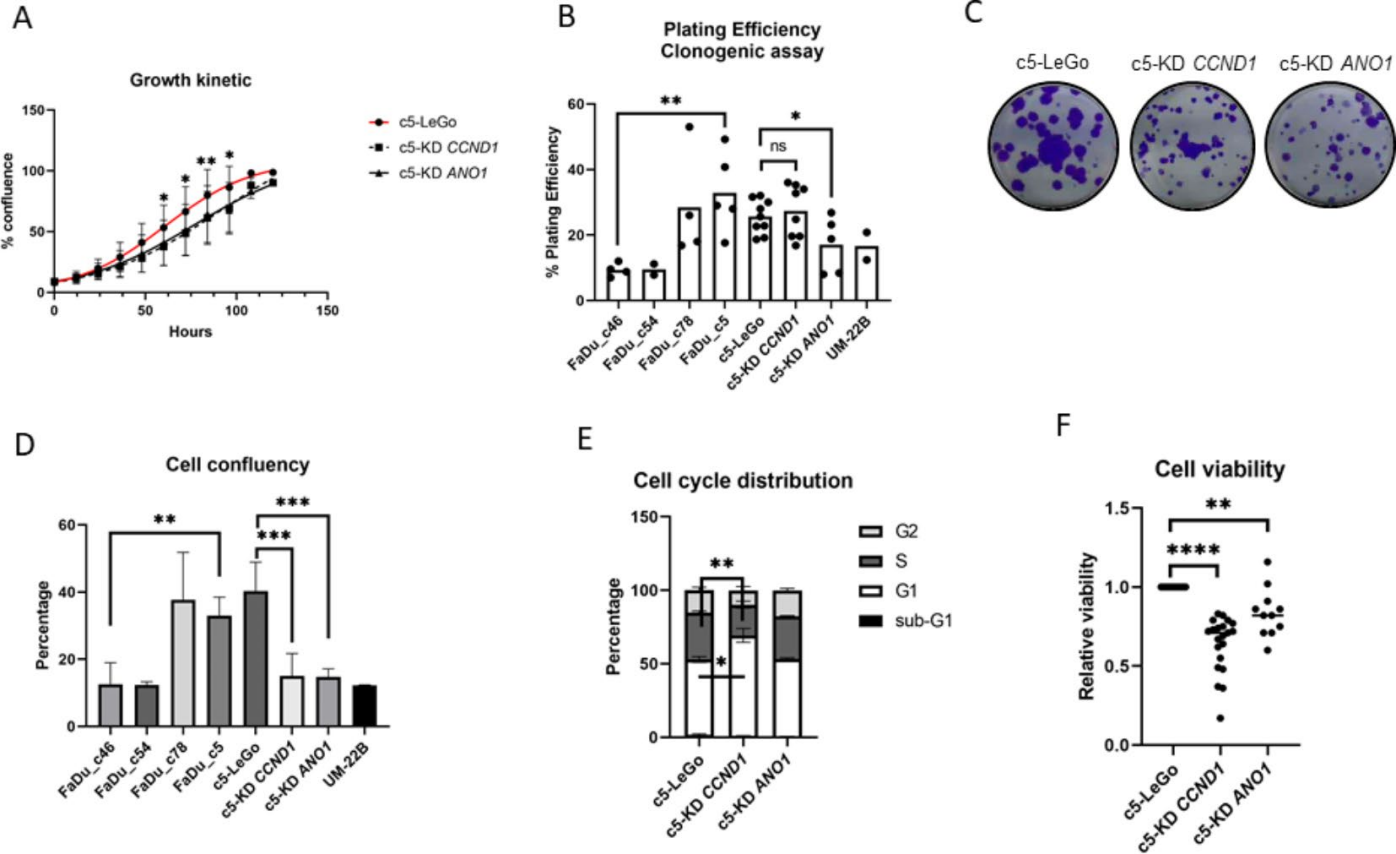
Functional characterization of *CCND1* and *ANO1*KD. (**A**)*CCND1* and *ANO1* KD significantly reduced the cell growth kinetic of FaDu compared to the control LeGo; (**B**) The plating efficiency is affected on *ANO1* KD but not *CCND1* KD, although the cell confluency is impaired for both KD (**C, D**); (**E**)*CCND1* KD resulted in G0/G1 phase arrest and a simultaneous reduction of the S-phase fraction. Bars represent ± SEM from three independent experiments; (**F**) The knockdown of *CCND1* and *ANO1* significantly reduce the cell metabolic activity, measured by MTT assay.

To confirm the functional impact of the common changes in DNA synthesis and cell cycle signaling pathways upon *CCND1* and *ANO1* KD, we analyzed growth kinetics as well as the cell cycle distribution. Cell growth was impaired by either KD (Fig. 3A). Interestingly, *ANO1* but not *CCND1* KD reduced the cells’ capacity to form colonies, resulting in a significantly lower plating efficiency compared to control cells (Fig. 3B). Although *CCND1* KD did not affect the plating efficiency, gene silencing reduced proliferation, leading to a decrease in the confluence of 2D monolayer cultures (Fig. 3C-D). Furthermore, *CCND1* and *ANO1* KD in the FaDu^res^ subclone c5 lowered its growth rate to a level similar to FaDu^sens^ subclones (c46, c54) (Fig. 3D). The cell cycle was dysregulated in *CCND1* KD cells, with a G1 arrest (+ 34% compared to LeGo) and a decrease in the S phase (-35% compared to LeGo) (Fig. 3E), indicating that *CCND1* KD hindered cell proliferation by blocking the G1/S transition. However, the gene enrichment analysis from the Reactome dataset demonstrated enrichment of pathways such as “S phase” and “Synthesis of DNA” observed in the upregulated protein expression in both KD (Fig. 2G). This could be explained by a compensatory mechanism upon gene silencing, leading to upregulation of other proteins involved in the same pathways, such as CKS1B (10.69-fold), Myc (3.83-fold), CDK2 (4.43-fold), LIG1 (3.5-fold), POLA1/2 (3.21-fold), CDC45 (5.53-fold) or ORC1/6 (2.32- and 33.96-fold respectively). On the contrary, *ANO1* KD did not significantly affect the cell cycle, though it did decrease the growth rate of cells. We also observed a decrease in metabolic activity of 35% for *CCND1* KD and 17% for *ANO1* KD (Fig. 3F). Corroborating the proteomic data, the results from the KD phenotype analysis confirmed the common role of Cyclin D1 and Ano1 in cell growth and viability.

### Overexpression of Ano1, but not cyclin D1, contributes to both radio- and CDDP resistance

**Fig. 4:**
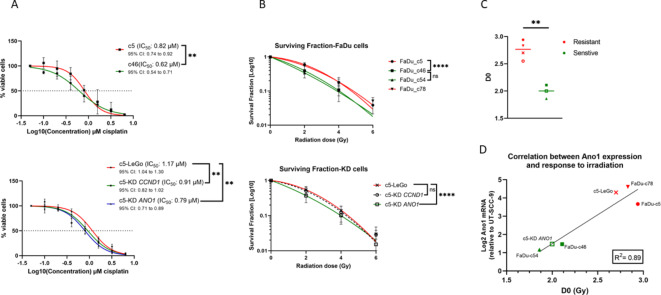
*ANO1* silencing resensitizes cells to DNA damaging treatment (radiation and CDDP). (**A,B**) Dose response curves to CDDP treatment (**A**) and radiation (**B**) in parental cells (upper) and after gene silencing (lower panels). Bars represent ± SEM from at least three independent experiments; (**C**) Classification of cells according to their sensitivity to radiation based on their D0. Symbols for cell lines correspond to those in B; (**D**) Correlation between Ano1 mRNA expression and radiosensitivity (D0).

Given that gene enrichment analysis revealed strong upregulation of DNA damage response signaling following *CCND1* and *ANO1* KD, we next examined the effect of KD on sensitivity to CDDP and radiation, both of which induce DNA damage. As expected from our previous study, FaDu c5 cells were less sensitive to CDDP (IC_50_: 0.82 µM) than FaDu c46 (IC_50_: 0.62 µM) (Fig. 4A). *CCND1* (IC_50_: 0.91 µM) and *ANO1* (IC_50_: 0.79 µM) KD slightly but significantly increased sensitivity to CDDP compared to control cells (LeGo: IC_50_: 1.17 µM) (Fig. 4A).

To assess radiosensitivity, we performed clonogenic survival assays, plotted survival curves based on the linear-quadratic model, and calculated D0 values for each cell line. The FaDu^res^ models showed a D0 value 45% higher than that of the FaDu^sens^ models (Fig. 4B and Supplementary Table 1; D0: 2.89 on average compared to 1.98), consistent with our previous findings^16^. We observed a moderate but significant increase in radiosensitivity when *ANO1* was silenced (Supplementary Table 1; D0: 2), whereas *CCND1* KD resulted in no major difference (Supplementary Table 1; D0: 2.55) compared to the vector control (Supplementary Table 1; D0: 2.70) (Fig. 4B). Using a cutoff of D0 ≥ 2.5, we identified two distinct radiation response groups: a resistant group (FaDu c5, c78, c5-LeGo, c5-KD *CCND1*) and a sensitive group (FaDu c46, c54, c5-KD *ANO1*; Fig. 4C). Notably, the cells with the most radiosensitive phenotype were those with the lowest Ano1 expression levels (Fig. 4D).

### Interference of cyclin D1 and Ano1 with DNA repair kinetics

**Fig. 5:**
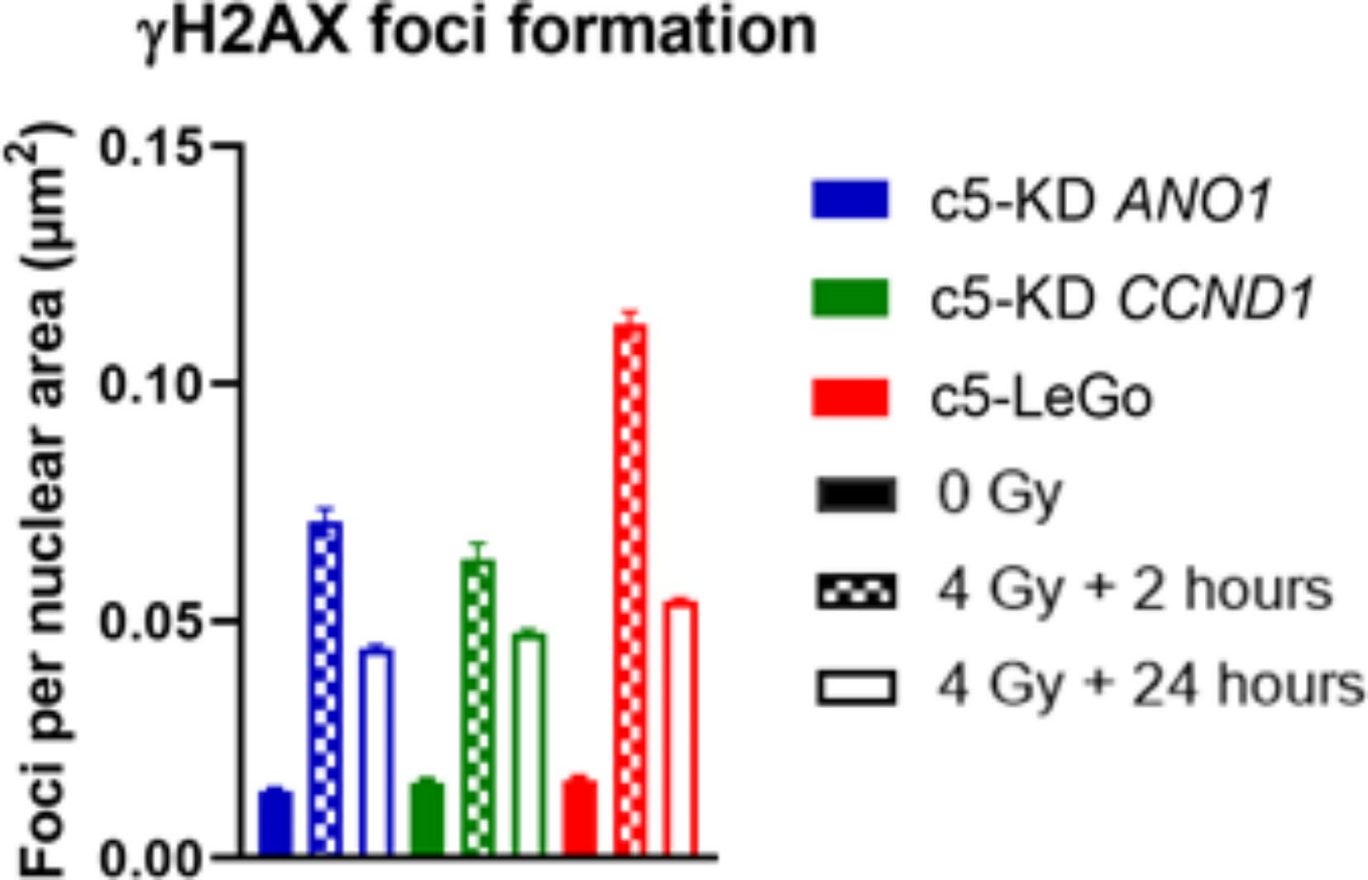
Kinetics of DNA repair determined by the γH2AX foci assay. Bars represent the average number of γH2AX foci per nuclear area (µm^2^) at 0 Gy and after irradiation with 4 Gy, measured at 2 h and 24 h post irradiation.

Gene enrichment analysis of the proteomics dataset identified changes in DNA damage repair genes commonly associated with *CCND1* and *ANO1* KD. To explore this further, we performed a comparative analysis of gene expression levels in TCGA HNSCC subgroups, stratified by Cyclin D1 and Ano1 expression. This analysis revealed significantly higher expression of genes involved in homologous recombination (BRCA1, BRCA2, Rad51B) in Ano1-overexpressing tumors, while Cyclin D1-overexpressing cells showed significantly elevated levels of XRCC6, a gene involved in non-homologous end-joining (Supplementary Table 2). Given these findings, we investigated whether silencing of *CCND1* or *ANO1* affected DNA repair in the CDDP-resistant FaDu model. Cell cycle analysis revealed only slight changes after irradiation, except in c5-LeGo, which showed an increase in G1 phase (Supplementary Figure S3). To assess DNA repair efficacy, we used the γH2AX foci assay to quantify DNA double-strand breaks (DSBs) at 2- and 24-hours following irradiation. At baseline, comparable numbers of γH2AX foci were detected in all cell lines. Two hours post irradiation, foci resolution was more advanced in *CCND1* KD and *ANO1* KD cells compared to vector control cells. By 24 h post irradiation, the number of residual foci had reached similar level across all three cell lines, with no major difference between *CCND1* and *ANO1* KD (Fig. 5).

###  Pre-treatment with palbociclib does not radiosensitize HPV-ve cells with high cyclin D1 level

**Fig. 6:**
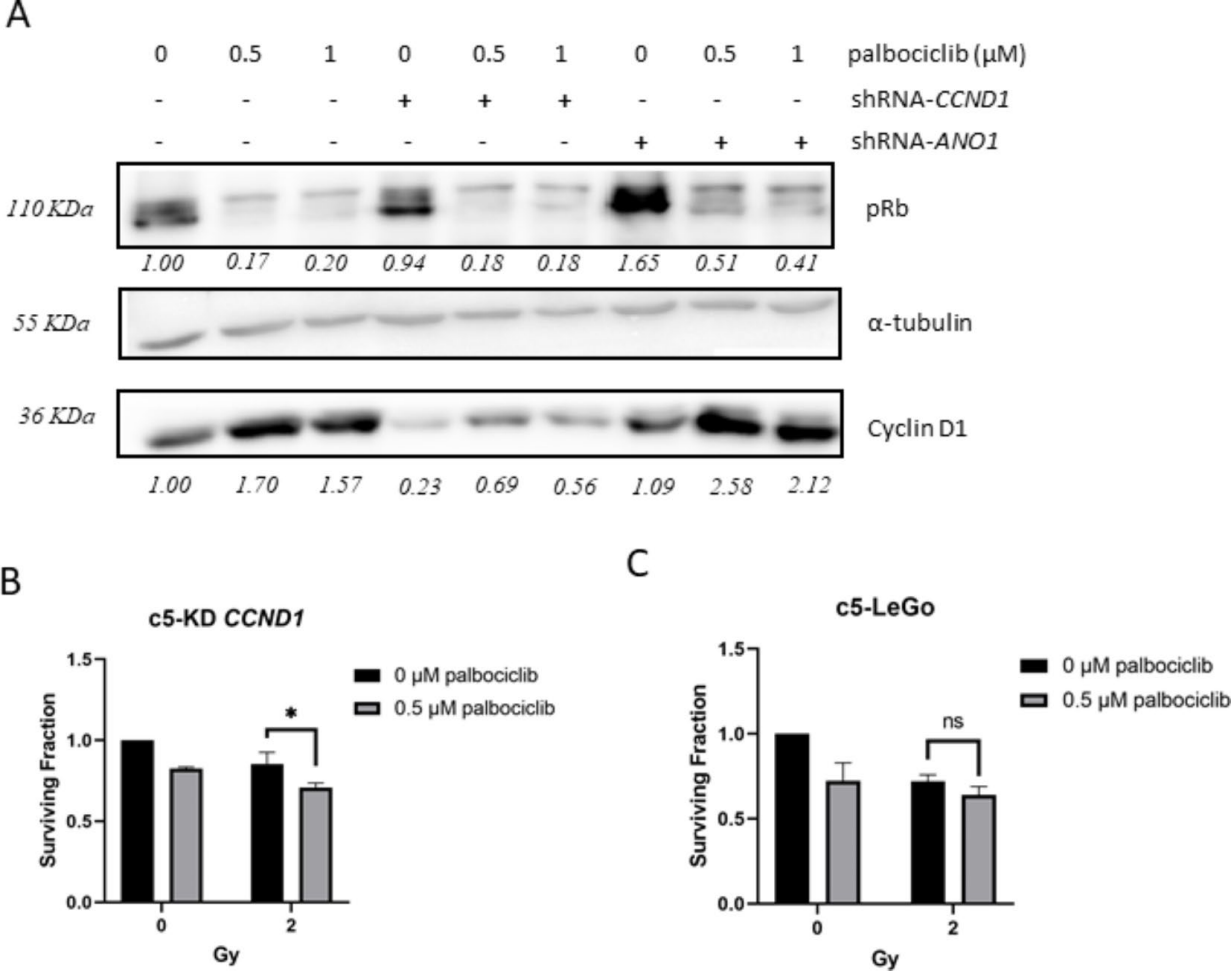
CDK4/6 inhibition does not radiosensitize cells with high expression of Cyclin D1. (**A**) Western blot analysis revealed down-regulation of pRb and upregulation of Cyclin D1 by palbociclib. Blot lanes have been cropped from the same gel. Original blots are presented in Supplementary Image S1; (**B-C**) Pretreatment with palbociclib (0.5 µM for 6 h) radiosensitized cells upon *CCND1* silencing (**B**) but not vector control cells (**C**). Bars represent ± SEM from at least three independent experiments.

To confirm Cyclin D1’s lack of involvement in radioresistance, we tested the CDK4/6 inhibitor palbociclib in combination with radiation. FaDu cells were treated with 0.5 µM and 1 µM of palbociclib for a short period of 24 h to minimize direct effects on cell viability. Palbociclib effectively inhibited Cyclin D1 signaling, as evidenced by a significant decrease in pRb levels (Fig. 6A). At higher doses, palbociclib reduced cell viability, regardless of Cyclin D1 expression levels (Supplementary Figure S4A). However, preincubation of cells with palbociclib (0.5 µM) for 6 h prior radiation slightly increased sensitivity to 2 Gy irradiation in *CCND1* KD cells (Fig. 6B), though it did not increase radiosensitivity of Cyclin D1-overexpressing cells (Fig. 6C). Similar results were obtained when palbociclib was combined with CDDP (Supplementary Figure S4B).

### Combination of PI3K and EGFR inhibition as a new treatment strategy for Cyclin D1^high^*-Ano1*^high^expressing HNSCC

**Fig. 7:**
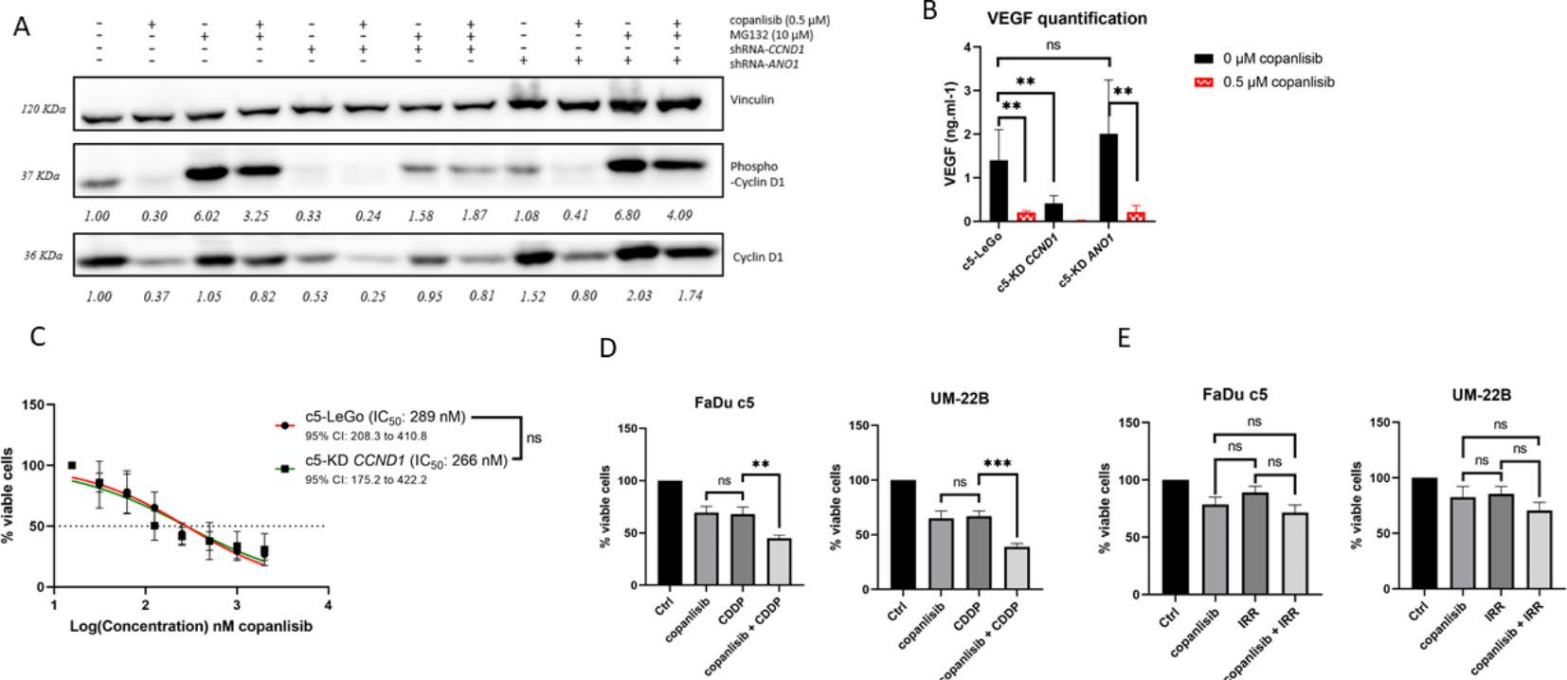
PI3K pathway inhibition with copanlisib leads to degradation of Cyclin D1 via the proteasome and sensitization to CDDP but not radiation. (**A**) Western blot representing downregulation of Cyclin D1 upon gene silencing or after treatment of 0.5 µM copanlisib for 24 h. This effect was reversed by treatment of the proteasome inhibitor MG132 (10 µM for 4 h). Samples were derived from the same experiment, and gels and blots were processed in parallel. Original blots are presented in Supplementary Image S2; (**B**) VEGF levels decreased upon copanlisib treatment (0.5 µM for 24 h); (**C**) Dose-response curves for copanlisib; (**D-E**) FaDu c5 and UM-22B, two HNSCC cell lines overexpressing Cyclin D1 were pretreated for 24 h with copanlisib before CDDP treatment (**D**) or irradiation with 4 Gy (**E**). Viability assay by MTT was done after 72 h (**D**) or 48 h (**E**). Bars represent ± SEM from at least three independent experiments.

The lack of a sensitizing effect of CDK4/CDK6 inhibition could be due to the observed increase in Cyclin D1 expression levels following palbociclib treatment (Fig. 6A). Thus, another strategy to downregulate Cyclin D1 signaling was tested. For this, we treated cells with copanlisib, a PI3-kinase inhibitor that reactivates GSK3ß (glycogen synthase kinase 3ß), thereby increasing phosphorylation of Cyclin D1 and its subsequent proteasomal degradation^18^ (see schematic representation of signaling pathways in Supplementary Figure S5). Indeed, copanlisib treatment decreased Cyclin D1 expression to levels similar to those achieved by gene silencing with shRNA, an effect that was reversible by the proteasome inhibitor MG-132 (Fig. 7A). To validate the use of copanlisib for blocking Cyclin D1 signaling, we performed an ELISA assay to quantify VEGF levels, which are known to be downregulated upon *CCND1* KD. Treatment with 0.5 µM copanlisib for 24 h reduced VEGF levels in all cell lines to a level comparable to that observed in *CCND1* knockdown cells (Fig. 7B). Of note, no substantial distinction in the IC₅₀ value was observed when cells were treated with copanlisib as a monotherapy, regardless of Cyclin D1 expression levels (Fig. 7C; IC_50_ value of c5-KD *CCND1*: 266 nM, compared to 289 nM for c5-LeGo). Interestingly, copanlisib used prior to CDDP had an additive effect in decreasing cell viability (Fig. 7D), whereas no additional effect was observed when combining copanlisib with radiation (Fig. 7E). These findings again confirm the role of Cyclin D1 in CDDP- but radioresistance in Cyclin D1-overexpressing HNSCC cells.

**Fig. 8:**
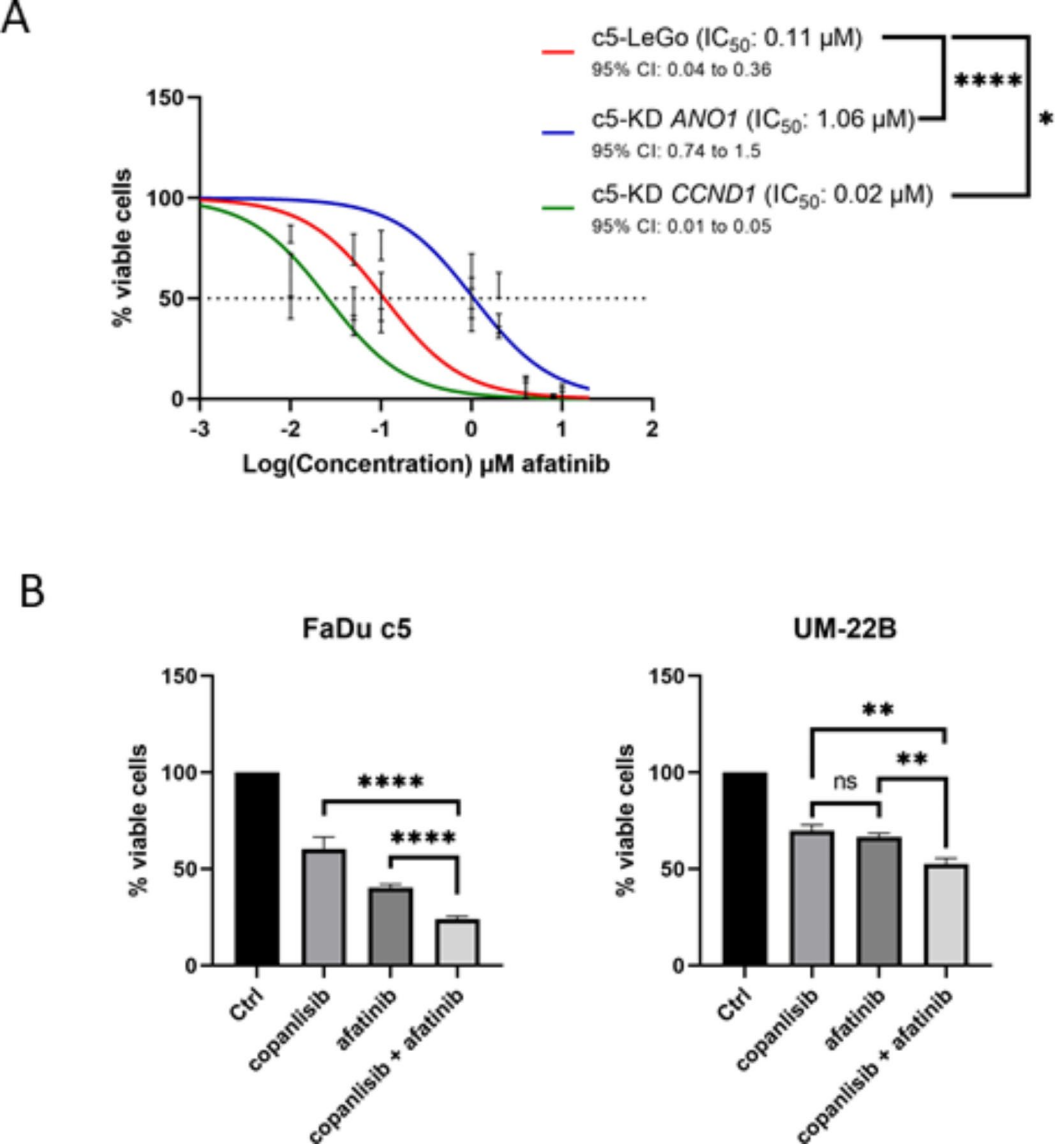
Additive inhibitory effect of copanlisib and afatinib in Cyclin D1^high^Ano1^high^cells. (**A**) Dose-response curves of afatinib treatment in vector control (LeGo) and KD cells; (**B**) FaDu c5 or UM-SCC-22B cells were treated with 0.15 µM of copanlisib for 24 h before addition of afatinib for 48 h. Bars represent ± SEM from at least three independent experiments.

We further investigated the effect of *CCND1* or *ANO1* gene silencing on the sensitivity to EGFR inhibition. High Cyclin D1 expression negatively affected sensitivity to afatinib (Fig. 8A). In contrast, high Ano1 expression, as previously reported^10^, increased sensitivity to afatinib, an effect that was reversed by silencing *ANO1*. We hypothesized that combining copanlisib (which lowers Cyclin D1 levels) with afatinib (which is more active in Ano1^high^ cells) might be a promising therapeutic strategy for 11q13 amplified HNSCC tumors. Treatment of FaDu c5 and UM-22B cells with copanlisib for 24 h before adding afatinib treatment enhanced their sensitivity compared to treatment with either drug alone (Fig. 8B).

## Discussion

Here, using the TCGA HNSCC dataset, we showed that overexpression of Ano1, even in the absence of high Cyclin D1 expression, significantly contributes to poor outcome after standard treatment in HPV-ve HNSCC tumors. We could validate these results in a Charité patient cohort uniformly treated with CDDP-based RCtx (*n* = 34), thereby identifying the *ANO1* gene as one of the master drivers of resistance to CDDP-based RCTx in patients with 11q13 amplifications. We also confirmed that high Ano1 expression levels were directly connected to a lower sensitivity to ionizing radiation. To our knowledge, this is the first study to establish a link between Ano1 expression and response to radiation in HNSCC.

Our results from the genetic perturbation of a CDDP-resistant HNSCC cell line model further support a direct role of Ano1 in mediating tumor progression and drug resistance. Knockdown of *ANO1* in a CDDP-resistant FaDu model resulted in decreased cell viability and growth, consistent with previous findings^19,20^. Moreover, cells were significantly re-sensitized to CDDP upon gene silencing. The observed chemoresistance may be attributed to low levels of the pro-apoptotic protein Bim in Ano1-overexpressing cells, impairing CDDP-induced apoptosis^21^ and increase drug efflux by lysosomal exocytosis, thereby significantly lowering intracellular CDDP concentrations^22^. To reverse this effect, the lysosomal inhibitor hydroxychloroquine (HCQ) has been shown to enhance the efficacy of cisplatin in the treatment of Ano1-overexpressed HNSCC^22^.

As first described by Bill et al.^10^, Ano1 forms a functional complex with EGFR, thereby stabilizing its expression. EGFR expression levels have been linked to radioresistance^23,24^, and a phase III trial showed improved overall survival in HNSCC patients treated with the EGFR-inhibitor cetuximab plus radiotherapy^25^. Thus, the increased sensitivity to irradiation observed in *ANO1* KD cells could be explained by the dysregulation of EGFR signaling. The lower radiosensitivity of Ano1-overexpressing cells may also result from their enhanced capacity to repair radiation-induced DNA damage, as shown by the higher expression of genes involved in homologous recombination (BRCA1, BRCA2, RAD51B).

Furthermore, the proteome analysis in the CDDP-resistant FaDu model in our study revealed that *ANO1* KD leads to downregulation of XPA, a NER-associated DNA repair gene, and HOXB7, a homeobox DNA-binding protein, both of which have already been associated with radioresistance in HNSCC^26,27^. However, our analysis of the DSB repair also showed improved resolution of γH2AX foci at 2 h post-irradiation, and similar levels of residual γH2AX foci at 24 h upon *ANO1* gene silencing, indicating a difference in the kinetics rather than the efficacy of the DNA repair processes, depending on Ano1 expression levels. Certainly, a more thorough mechanistic analysis of the DNA repair processes will be required to fully understand the role of Ano1 in radioresistance.

In contrast to the radiosensitizing effect of *ANO1* KD, no significant change in radiosensitivity was observed when *CCND1* was silenced. This is in contrast to previous findings of Shintani et al.^28^ reporting enhanced radiosensitivity of Cyclin D1-overexpressing oral SCC cell lines, and improved outcome after RTx in oral SCC patients^28^. However, an experimental proof of a causal relationship between Cyclin D1 overexpression and increased radiosensitivity was not provided by this study, leaving open the possibility that other genes co-regulated with Cyclin D1 (e.g. Ano1 according to our data) might be responsible for the observed association.

As expected, *CCND1* silencing per se lowered the fraction of actively cycling cells in our model, and the arrest in G1 was even more pronounced upon irradiation. The decelerating effects of the *CCND1* KD on the cell cycle leaves cells more time for DNA repair, as reported from comparative analysis of quiescent and proliferating cells^29,30^. This could potentially counteract a potential radiosensitizing effect of *CCND1* silencing in our model system.

Alternatively, a decrease in Cyclin D1 expression levels alone may not be sufficient to re-sensitize cells to radiation. However, combining palbociclib with radiation, as demonstrated by Gottgens et al.^31^, increased the radiosensitivity of the parental FaDu cell line. This effect was mediated through the downregulation of pRb and repression of the HR pathway genes RAD51 and BRCA1, eventually leading to persistent DNA damage. Similar findings were observed in HPV-ve HNSCC cell lines without 11q13 amplification^32^. In our study, palbociclib pretreatment also enhanced radiosensitivity only in non-amplified cells. Comparable absence of additional effects was seen with the combination of palbociclib and cisplatin in nasopharyngeal carcinoma^33^, in HPV-ve HNSCC^34^, and in studies involving abemaciclib and cisplatin, where ambivalent results were reported in HNSCC^35^.

Given that Cyclin D1 may also engage in extra-nuclear signaling, we tested copanlisib, a PI3K inhibitor that induces Cyclin D1 downregulation through proteasomal degradation. Similar to palbociclib, copanlisib did not sensitize Cyclin D1-overexpressing cells to radiation. However, it showed an additive effect when combined with cisplatin. These data support the use of a PI3K inhibitor as a concurrent treatment to pharmacologically downregulate Cyclin D1 prior to platinum therapy, a combinatory strategy whose feasibility has already been demonstrated in Phase I studies in HNSCC patients^36^.

PI3K closely interacts with its upstream effector EGFR, via the regulation of the RAS pathway^37^. Because of its association with reduced survival, EGFR represents a compelling molecular target for the treatment of HNSCC. Our findings demonstrated that afatinib, a pan-EGFR inhibitor, noticeably reduced cell viability in our Ano1^high^ and Cyclin D1^low^ models. We therefore hypothesized that the use of copanlisib to decrease Cyclin D1 level prior to afatinib may improve the efficacy of afatinib treatment in overexpressed cells. The in vitro results demonstrated that the combination of the two treatments had an additive effect. In HNSCC HPV + ve in vitro cells, the combination of copanlisib and afatinib showed promising results by suppressing cell proliferation and survival^38^. A trial to assess the clinical relevance of this combination would provide a treatment option for the unfavourable HPV-ve subset. Assessment of efficacy and toxicity of combined treatment with EGFR and PI3K inhibitors has revealed varied results. For example, targeting EGFR ligand binding with cetuximab in HNSCC showed high toxicity when combined with copanlisib (NCT02822482)^39^ but was well-tolerated when combined with alpelisib (NCT02282371 ^40^, NCT01602315 ^41^) and showed antitumor activity (NCT02282371)^40^. A clinical trial investigating the combination of a PI3K inhibitor with a first-generation EGFR tyrosine kinase inhibitor (TKi) revealed antitumor activity but also late toxicity, as seen with gefitinib and buparlisib in NSCLC (NCT01570296)^42^. To our knowledge, no clinical trials have assessed the safety of combining the second-generation EGFR-TKi afatinib with copanlisib. However, preclinical studies demonstrated synergistic inhibition of xenograft tumor growth with this combination, without significant impact on body weight in mice^43^. These findings suggest that second-generation EGFR-TKIs combined with PI3K inhibitors may represent a promising therapeutic strategy deserving further clinical investigation.

Our study has two major limitations. First, the majority of the functional studies were conducted in 2D cultures, which lack critical cell-to-cell interactions. Although we utilized 3D cultures that better replicate cell-cell and cell-extracellular matrix interactions, these were only employed in drug testing experiments and not for radiosensitivity assays or mass spectrometry analysis. Another limitation is the use of one CDDP-resistant 11q13-amplified model for shRNA silencing experiments. While we consider this model ideal for studying mechanisms of primary and acquired resistance^16^, it may have limited our ability to identify additional resistance phenotypes in Ano1- and Cyclin D1-overexpressing cells. Thus, results from drug screens were validated in another 11q13 amplified cell line model. Further studies in patient-derived primary models will be necessary and should include the testing of novel therapeutic strategies for this challenging group of tumors.

## Conclusions

In this study, we corroborate the important role of Cyclin D1 and Ano1 in cell viability, proliferation and their impact on therapy resistance in HNSCC. We demonstrated that Cyclin D1 and Ano1 overexpression contributes to CDDP resistance. We revealed for the first time the role of Ano1 in modulating sensitivity to ionizing radiation in HPV-ve HNSCC. Results from drug testing suggest a therapeutic potential of dual PI3K/EGFR inhibition as well as a PI3K inhibitor-CDDP combinations for HPV-ve HNSCC patients exhibiting high levels of Cyclin D1 and Ano1. Validation of our findings in patient-derived organoids and xenografts is warranted.

## Electronic supplementary material

Below is the link to the electronic supplementary material.

Supplementary Material 1

## Data Availability

Data availability: All data generated or analyzed during this study are included in this published article (and its Supplementary Information files).

## References

[1] Sung, H. et al. Global Cancer statistics 2020: GLOBOCAN estimates of incidence and Mortality Worldwide for 36 cancers in 185 countries. *CA Cancer J. Clin.* **71**, 209–249. 10.3322/caac.21660 (2021). 33538338 10.3322/caac.21660

[2] Mody, M. D., Rocco, J. W., Yom, S. S., Haddad, R. I. & Saba, N. F. Head and neck cancer. *Lancet* **398**, 2289–2299. 10.1016/S0140-6736(21)01550-6 (2021). 34562395 10.1016/S0140-6736(21)01550-6

[3] Mehanna, H. et al. Prognostic implications of p16 and HPV discordance in oropharyngeal cancer (HNCIG-EPIC-OPC): A multicentre, multinational, individual patient data analysis. *Lancet Oncol.* **24**, 239–251. 10.1016/S1470-2045(23)00013-X (2023). 36796393 10.1016/S1470-2045(23)00013-X

[4] Lacas, B. et al. Meta-analysis of chemotherapy in head and neck cancer (MACH-NC): An update on 107 randomized trials and 19,805 patients, on behalf of MACH-NC Group. *Radiother Oncol.* **156**, 281–293. 10.1016/j.radonc.2021.01.013 (2021). 33515668 10.1016/j.radonc.2021.01.013 PMC8386522

[5] Ramos-Garcia, P. et al. Relevance of chromosomal band 11q13 in oral carcinogenesis: An update of current knowledge. *Oral Oncol.* **72**, 7–16. 10.1016/j.oraloncology.2017.04.016 (2017). 28797464 10.1016/j.oraloncology.2017.04.016

[6] Ruiz, C. et al. Enhanced expression of ANO1 in head and neck squamous cell carcinoma causes cell migration and correlates with poor prognosis. *PLoS One*. **7**, e43265. 10.1371/journal.pone.0043265 (2012). 22912841 10.1371/journal.pone.0043265 PMC3422276

[7] Lundberg, A. S. & Weinberg, R. A. Control of the cell cycle and apoptosis. *Eur. J. Cancer*. **35**, 1886–1894. 10.1016/s0959-8049(99)00292-0 (1999). 10711231 10.1016/s0959-8049(99)00292-0

[8] Yang, Y. D. et al. TMEM16A confers receptor-activated calcium-dependent chloride conductance. *Nature* **455**, 1210–1215. 10.1038/nature07313 (2008). 18724360 10.1038/nature07313

[9] Huang, F. et al. Studies on expression and function of the TMEM16A calcium-activated chloride channel. *Proc. Natl. Acad. Sci. U S A*. **106**, 21413–21418. 10.1073/pnas.0911935106 (2009). 19965375 10.1073/pnas.0911935106 PMC2781737

[10] Bill, A. et al. ANO1/TMEM16A interacts with EGFR and correlates with sensitivity to EGFR-targeting therapy in head and neck cancer. *Oncotarget* **6**, 9173–9188. 10.18632/oncotarget.3277 (2015). 25823819 10.18632/oncotarget.3277 PMC4496210

[11] Grandis, J. R. & Tweardy, D. J. Elevated levels of transforming growth factor alpha and epidermal growth factor receptor messenger RNA are early markers of carcinogenesis in head and neck cancer. *Cancer Res.* **53**, 3579–3584 (1993). 8339264

[12] Rubin Grandis, J. et al. Levels of TGF-alpha and EGFR protein in head and neck squamous cell carcinoma and patient survival. *J. Natl. Cancer Inst.* **90**, 824–832. 10.1093/jnci/90.11.824 (1998). 9625170 10.1093/jnci/90.11.824

[13] Ang, K. K. et al. Impact of epidermal growth factor receptor expression on survival and pattern of relapse in patients with advanced head and neck carcinoma. *Cancer Res.* **62**, 7350–7356 (2002). 12499279

[14] Okuyama, K. & Yanamoto, S. TMEM16A as a potential treatment target for head and neck cancer. *J. Experimental Clin. Cancer Res.* **41**, 196. 10.1186/s13046-022-02405-2 (2022). 10.1186/s13046-022-02405-2 PMC9172006 35668455

[15] Carles, A. et al. Head and neck squamous cell carcinoma transcriptome analysis by comprehensive validated differential display. *Oncogene* **25**, 1821–1831. 10.1038/sj.onc.1209203 (2006). 16261155 10.1038/sj.onc.1209203

[16] Niehr, F. et al. Multilayered Omics-based analysis of a Head and Neck Cancer Model of Cisplatin Resistance reveals Intratumoral Heterogeneity and Treatment-Induced Clonal Selection. *Clin. Cancer Res.* **24**, 158–168. 10.1158/1078-0432.CCR-17-2410 (2018). 29061642 10.1158/1078-0432.CCR-17-2410

[17] Kordbacheh, F. & Farah, C. S. Molecular pathways and druggable targets in Head and Neck squamous cell carcinoma. *Cancers (Basel)*. 10.3390/cancers13143453 (2021). 10.3390/cancers13143453 PMC8307423 34298667

[18] VanArsdale, T., Boshoff, C., Arndt, K. T. & Abraham, R. T. Molecular pathways: Targeting the cyclin D-CDK4/6 Axis for Cancer Treatment. *Clin. Cancer Res.* **21**, 2905–2910. 10.1158/1078-0432.CCR-14-0816 (2015). 25941111 10.1158/1078-0432.CCR-14-0816

[19] Britschgi, A. et al. Calcium-activated chloride channel ANO1 promotes breast cancer progression by activating EGFR and CAMK signaling. *Proc. Natl. Acad. Sci. U S A*. **110**, E1026–1034. 10.1073/pnas.1217072110 (2013). 23431153 10.1073/pnas.1217072110 PMC3600458

[20] Duvvuri, U. et al. TMEM16A induces MAPK and contributes directly to tumorigenesis and cancer progression. *Cancer Res.* **72**, 3270–3281. 10.1158/0008-5472.CAN-12-0475-T (2012). 22564524 10.1158/0008-5472.CAN-12-0475-T PMC3694774

[21] Godse, N. R. et al. TMEM16A/ANO1 inhibits apoptosis Via downregulation of Bim expression. *Clin. Cancer Res.* **23**, 7324–7332. 10.1158/1078-0432.CCR-17-1561 (2017). 28899969 10.1158/1078-0432.CCR-17-1561 PMC5898434

[22] Vyas, A. et al. Lysosomal inhibition sensitizes TMEM16A-expressing cancer cells to chemotherapy. *Proc. Natl. Acad. Sci. U S A*. **119**, e2100670119. 10.1073/pnas.2100670119 (2022). 35286200 10.1073/pnas.2100670119 PMC8944912

[23] Bonner, J. A., Maihle, N. J., Folven, B. R., Christianson, T. J. & Spain, K. The interaction of epidermal growth factor and radiation in human head and neck squamous cell carcinoma cell lines with vastly different radiosensitivities. *Int. J. Radiat. Oncol. Biol. Phys.* **29**, 243–247. 10.1016/0360-3016(94)90269-0 (1994). 8195014 10.1016/0360-3016(94)90269-0

[24] Sheridan, M. T., O’Dwyer, T., Seymour, C. B. & Mothersill, C. E. Potential indicators of radiosensitivity in squamous cell carcinoma of the head and neck. *Radiat. Oncol. Investig*. **5**, 180–186. https://doi.org/10.1002/(SICI)1520-6823(1997)5:4<180::AID-ROI3>3.0.CO;2-U (1997). 10.1002/(SICI)1520-6823(1997)5:4<180::AID-ROI3>3.0.CO;2-U 9327497

[25] Bonner, J. A. et al. Radiotherapy plus Cetuximab for squamous-cell carcinoma of the head and neck. *N Engl. J. Med.* **354**, 567–578. 10.1056/NEJMoa053422 (2006). 16467544 10.1056/NEJMoa053422

[26] Todorovic, V. et al. Mechanisms of different response to ionizing irradiation in isogenic head and neck cancer cell lines. *Radiat. Oncol.* **14**, 214. 10.1186/s13014-019-1418-6 (2019). 31775835 10.1186/s13014-019-1418-6 PMC6882348

[27] Yuan, Z., Chen, D., Chen, X., Yang, H. & Wei, Y. Overexpression of HOXB7 protein reduces sensitivity of oral cancer cells to chemo-radiotherapy. *Cancer Gene Ther.* **23**, 419–424. 10.1038/cgt.2016.55 (2016). 27834359 10.1038/cgt.2016.55

[28] Shintani, S., Mihara, M., Ueyama, Y., Matsumura, T. & Wong, D. T. Cyclin D1 overexpression associates with radiosensitivity in oral squamous cell carcinoma. *Int. J. Cancer*. **96**, 159–165. 10.1002/ijc.1014 (2001). 11410884 10.1002/ijc.1014

[29] Dikomey, E. Induction and repair of DNA strand breaks in X-irradiated proliferating and quiescent CHO cells. *Int. J. Radiat. Biol.* **57**, 1169–1182. 10.1080/09553009014551271 (1990). 1971842 10.1080/09553009014551271

[30] Eckers, J. C. et al. Forkhead box M1 regulates quiescence-associated radioresistance of human head and neck squamous carcinoma cells. *Radiat. Res.* **182**, 420–429. 10.1667/RR13726.1 (2014). 25229973 10.1667/RR13726.1 PMC4221113

[31] Gottgens, E. L. et al. Inhibition of CDK4/CDK6 enhances radiosensitivity of HPV negative head and Neck squamous cell carcinomas. *Int. J. Radiat. Oncol. Biol. Phys.* **105**, 548–558. 10.1016/j.ijrobp.2019.06.2531 (2019). 31271827 10.1016/j.ijrobp.2019.06.2531

[32] Shrivastava, N. et al. CDK4/6 inhibition induces senescence and enhances Radiation Response by disabling DNA damage repair in oral cavity squamous cell carcinoma. *Cancers (Basel)*. 15. 10.3390/cancers15072005 (2023). 10.3390/cancers15072005 PMC10093103 37046664

[33] Xue, Z. et al. Therapeutic evaluation of palbociclib and its compatibility with other chemotherapies for primary and recurrent nasopharyngeal carcinoma. *J. Exp. Clin. Cancer Res.* **39**, 262. 10.1186/s13046-020-01763-z (2020). 33243298 10.1186/s13046-020-01763-z PMC7690146

[34] Gu, Z. et al. Palbociclib-based high-throughput combination drug screening identifies synergistic therapeutic options in HPV-negative head and neck squamous cell carcinoma. *BMC Med.* **20**, 175. 10.1186/s12916-022-02373-6 (2022). 35546399 10.1186/s12916-022-02373-6 PMC9097351

[35] Schoenwaelder, N. et al. Preclinical Head and Neck squamous cell carcinoma models for combined targeted therapy approaches. *Cancers (Basel)*. 10.3390/cancers14102484 (2022). 10.3390/cancers14102484 PMC9139292 35626088

[36] Day, D. et al. Phase I trial of alpelisib in combination with concurrent cisplatin-based chemoradiotherapy in patients with locoregionally advanced squamous cell carcinoma of the head and neck. *Oral Oncol.* **108**, 104753. 10.1016/j.oraloncology.2020.104753 (2020). 32464516 10.1016/j.oraloncology.2020.104753

[37] Zaryouh, H. et al. Recent insights in the PI3K/Akt pathway as a promising therapeutic target in combination with EGFR-targeting agents to treat head and neck squamous cell carcinoma. *Med. Res. Rev.* **42**, 112–155. 10.1002/med.21806 (2022). 33928670 10.1002/med.21806

[38] Yang, Z. et al. Simultaneously targeting ErbB family kinases and PI3K in HPV-positive head and neck squamous cell carcinoma. *Oral Oncol.* **131**, 105939. 10.1016/j.oraloncology.2022.105939 (2022). 35667295 10.1016/j.oraloncology.2022.105939

[39] Marret, G. et al. Phase I trial of copanlisib, a selective PI3K inhibitor, in combination with cetuximab in patients with recurrent and/or metastatic head and neck squamous cell carcinoma. *Invest. New. Drugs*. **39**, 1641–1648. 10.1007/s10637-021-01152-z (2021). 34322775 10.1007/s10637-021-01152-z

[40] Dunn, L. A. et al. A phase 1b study of Cetuximab and BYL719 (Alpelisib) concurrent with Intensity Modulated Radiation Therapy in Stage III-IVB Head and Neck squamous cell carcinoma. *Int. J. Radiat. Oncol. Biol. Phys.* **106**, 564–570. 10.1016/j.ijrobp.2019.09.050 (2020). 31678634 10.1016/j.ijrobp.2019.09.050 PMC9478566

[41] Razak, A. R. A. et al. A phase 1b/2 study of Alpelisib in Combination with Cetuximab in patients with recurrent or metastatic Head and Neck squamous cell carcinoma. *Target. Oncol.* **18**, 853–868. 10.1007/s11523-023-00997-z (2023). 37875771 10.1007/s11523-023-00997-z PMC10663259

[42] Tan, D. S. W. et al. A phase ib safety and tolerability study of a pan class I PI3K inhibitor buparlisib (BKM120) and gefitinib (gef) in EGFR TKI-resistant NSCLC. *J. Clin. Oncol.* **31**, 8107–8107. 10.1200/jco.2013.31.15_suppl.8107 (2013).

[43] Dan, H. et al. Evaluation of Co-Inhibition of ErbB Family and PI3K Kinases for HPV-Negative Head and Neck Squamous Cell Carcinoma. 10.20944/preprints202402.0503.v1 (Preprints, 2024).

