# Supplementary material for: High Ano1 expression as key driver of resistance to radiation and cisplatin in HPV-negative head and neck squamous cell carcinoma
Source: Sci Rep. 2025 Jan 10;15:1555. doi: 10.1038/s41598-025-85214-9 (PMC11718065; doi:10.1038/s41598-025-85214-9)

# High Ano1 expression as key driver of resistance to radiation and cisplatin in HPV-negative head and neck squamous cell carcinoma

**Solenne Bourdier[1], Anne-Sophie Fisch[1], Keziban Merve Alp[2], Ridhima Das[1], Philipp Mertins[2] and Ingeborg Tinhofer[1,3]***

[1] Charité – Universitätsmedizin Berlin, corporate member of Freie Universität Berlin and Humboldt-Universität zu Berlin, Department of Radiooncology and Radiotherapy, Charitéplatz 1, 10117 Berlin, Germany
[2] Max Delbrück Center for Molecular Medicine, Robert-Rössle-Str. 10, 13092 Berlin, Germany
[3] German Cancer Consortium (DKTK) Partner Site Berlin, and German Cancer Research Center (DKFZ), 69120 Heidelberg, Germany

***** Correspondence: Prof. Dr. Tinhofer-Keilholz. E-mail: ingeborg.tinhofer@charite.de

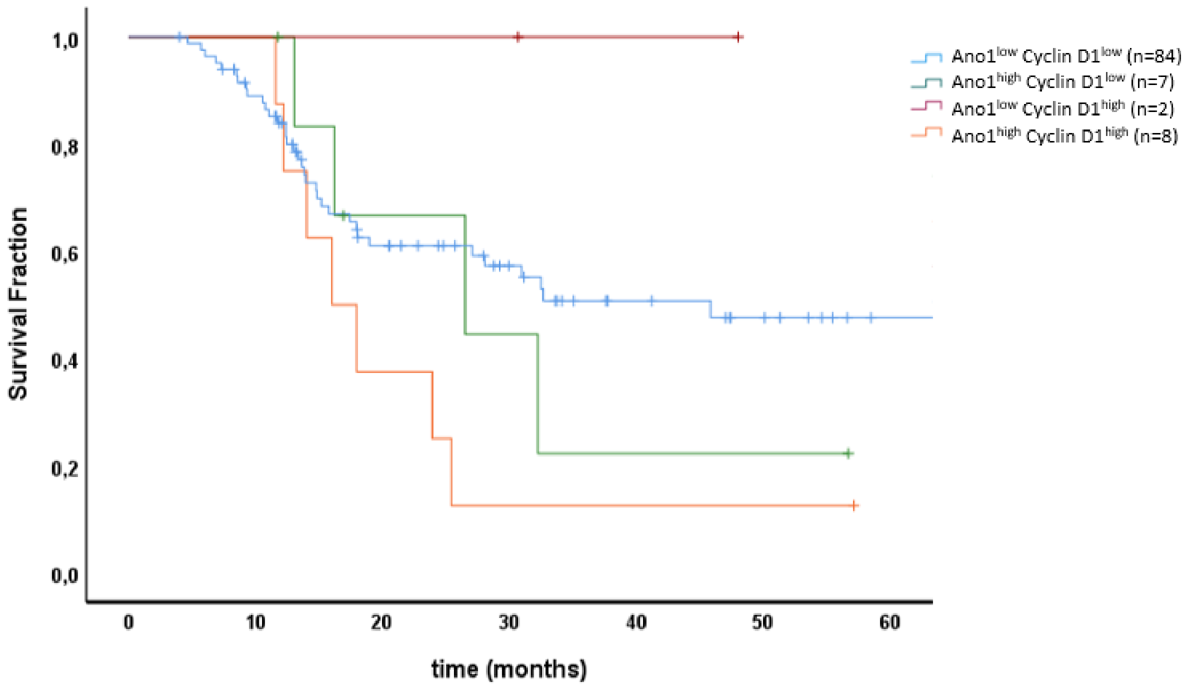


**Supplementary Figure S1**: Kaplan Meier curves of overall survival for locally advanced HPV-ve HNSCC patients treated with platinum-based RCTx (TCGA HNSCC PanCancer Atlas dataset; n=101) according to Ano1 and Cyclin D1 expression levels.

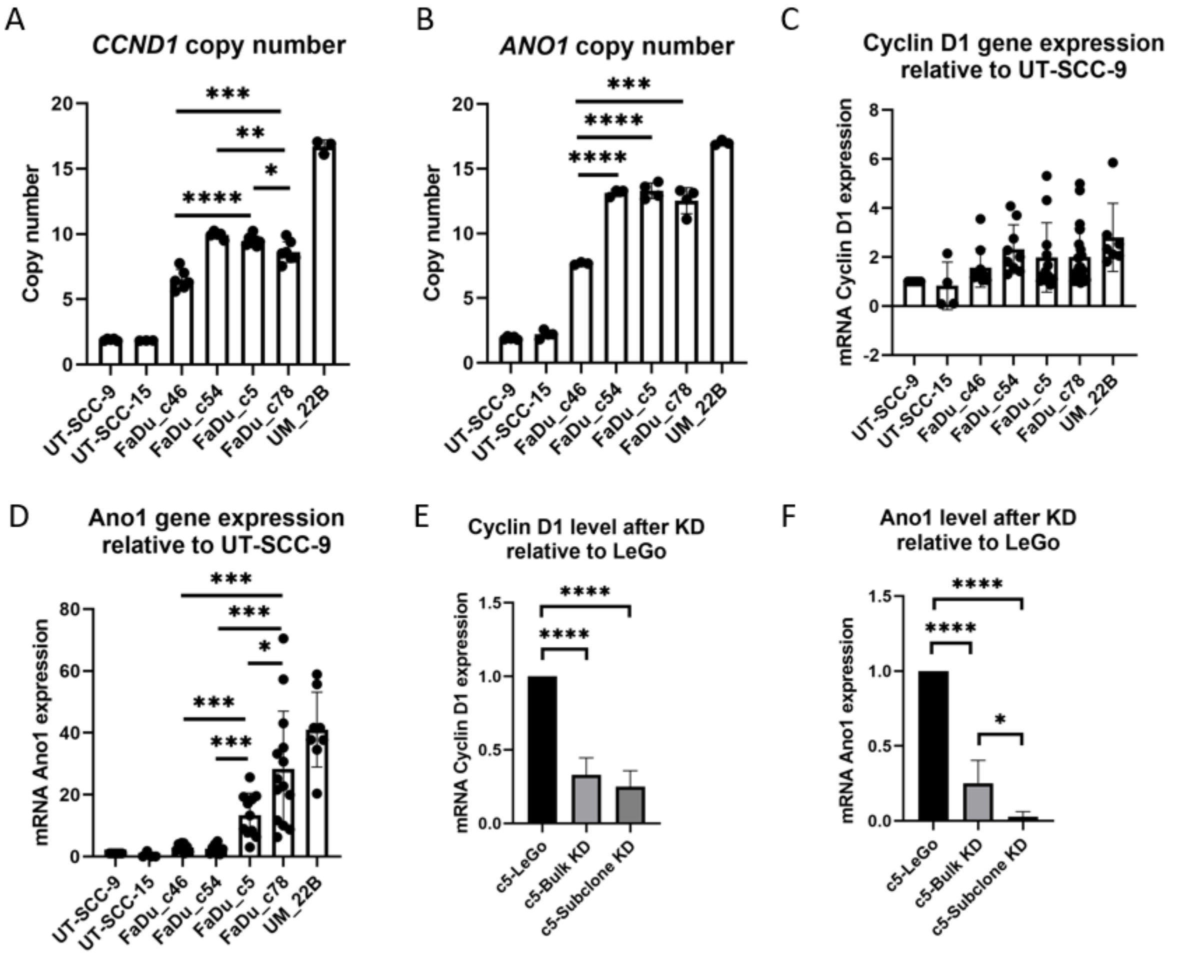


**Supplementary Figure S2: Genotype analysis of HNSCC cell lines. (A,B)** Droplet PCR measuring the copy number of *CCND1* **(A)** and *ANO1* **(B)** in 11q13 non-amplified HNSCC (UT-SCC-9, UT-SCC-15) and 11q13 amplified HNSCC cell lines (FaDu, UM-22B); **(C,D)** Gene expression of Cyclin D1 **(C)** and Ano1 **(D)**, relative to UT-SCC-9 was analyzed by qRT-PCR; **(E,F)** Efficacy of single-gene silencing compared to vector control (LeGo) was confirmed by qRT-PCR. Bars represent ±SEM from at least three independent experiments.

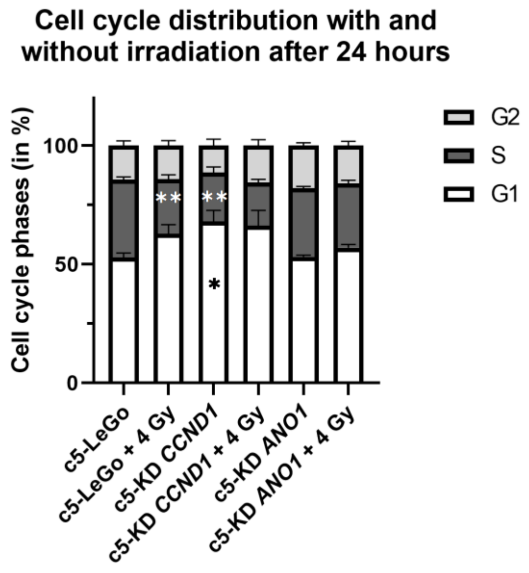


**Supplementary Figure S3: Effect on the cell cycle distribution of KD cells at 24 hours post-irradiation (4 Gy).** Stars represent the statistically significant changes in cell cycle distribution relative to c5-LeGo. Bars represent ±SEM from at least three independent experiments.

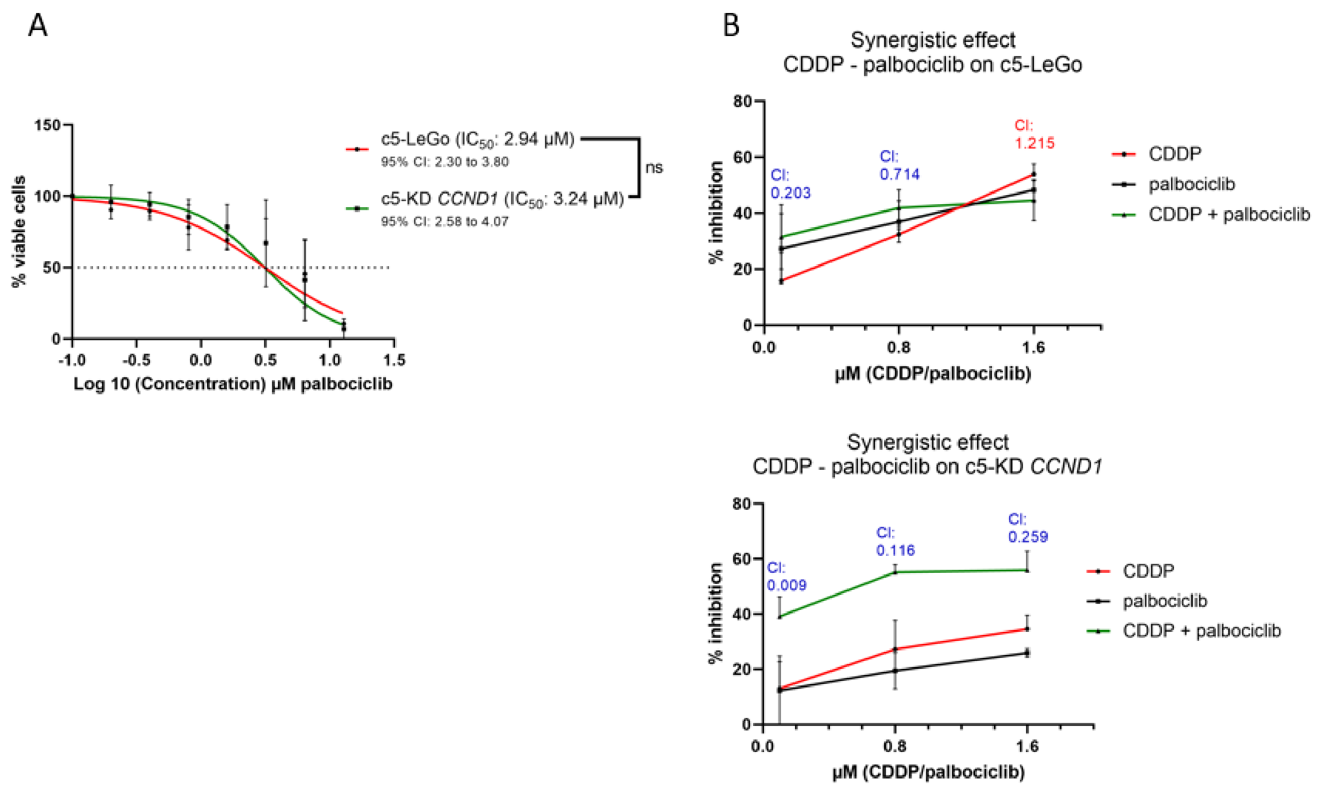


**Supplementary Figure S4: Effect of palbociclib on *CCND1* KD cells, alone or in combination with CDDP. (A)** $IC_{50}$ value of palbociclib. 2D cell culture were treated at a range concentration of 0-12.8 µM for 72 hours. Bars represent ±SEM from at least three independent experiments; **(B)** Synergistic effect of palbociclib and CDDP on c5-LeGo and c5-KD *CCND1* cells, analyzed by MTT cell viability assay. Cells were treated as a monotherapy at 0.1; 0.8 or 1.6 µM or in combination with a fixed dose of palbociclib (0.5 µM). Synergistic indexes were calculated from SynergyFinder+ where CI<1 shows a synergism and CI>1 an antagonism effect. Only the cells presenting low level of Cyclin D1 exhibit a synergistic effect of combined palbociclib and CDDP treatment.

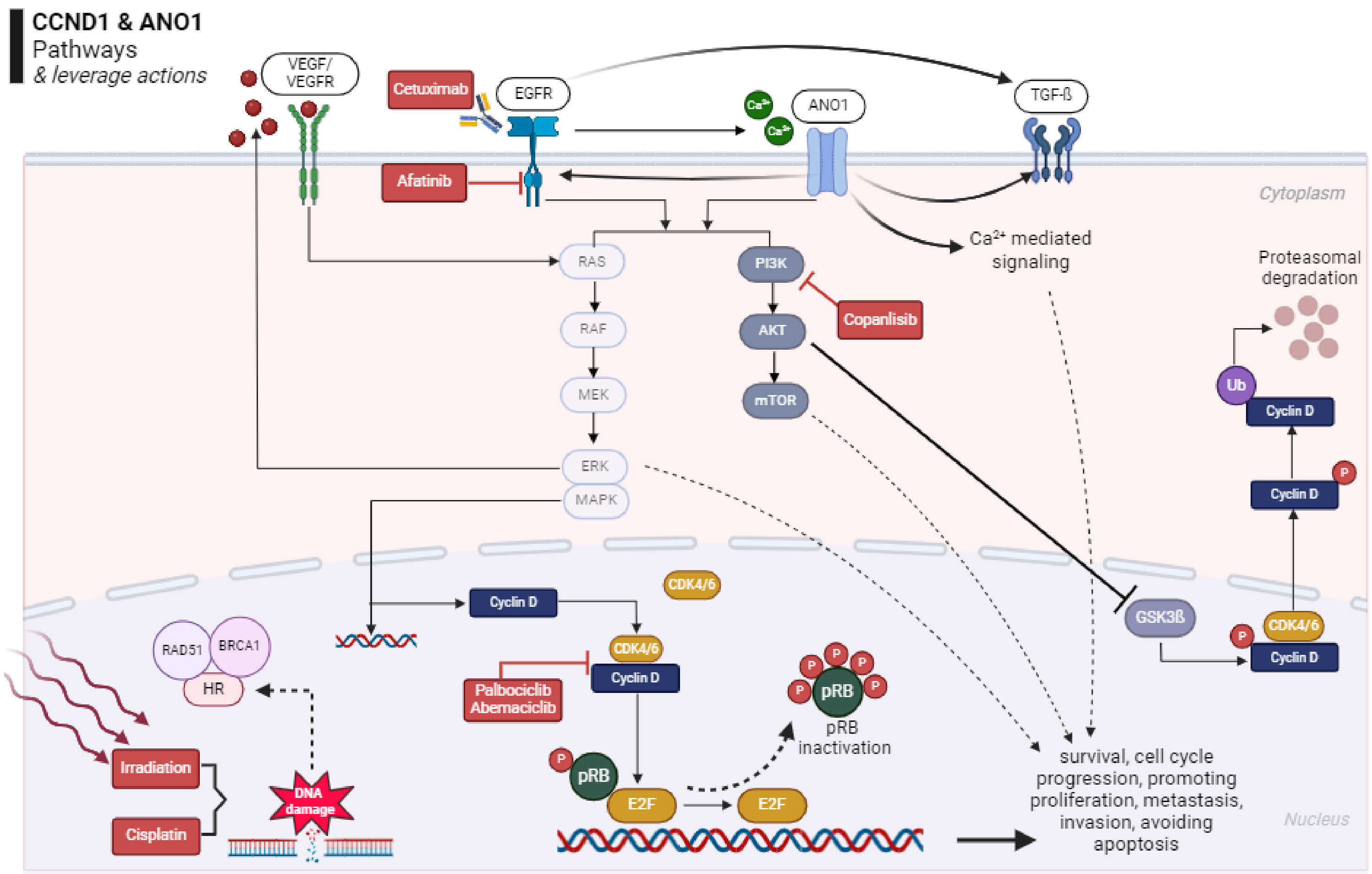


**Supplementary Figure S5: Cyclin D1 and Ano1 pathways and leverage actions for targeted therapies**. In the nucleus, Cyclin D1 forms a complex with CDK4/6 which initiates the phosphorylation of Retinoblastoma (pRB) leading to its inactivation and the release of E2F, an elongation factor that promotes the transcription of genes needed for cell cycle progression and cellular division. In the cytoplasm, Cyclin D1 is regulated by proteasome degradation, initiated by a phosphorylation mark induced by GSK3ß. However, high expression of PI3K down-regulates GSK3ß which in return impairs the phosphorylation of Cyclin D1. DNA damage generated by cisplatin or radiation are standard of care for HNSCC but the increase of chemo or radioresistance oblige to find other targetable pathways. The complex CDK4/6-Cyclin D1 could be inhibited by palbociclib and used to limit cell proliferation. Copanlisib, a potent PI3K inhibitor could be used to directly down-regulate Cyclin D1 via proteasomal degradation. Ano1 and EGFR form a strong interconnection and stabilize each other. Ano1 promotes proliferation, metastasis and invasion via RAS/RAF/MEK/MAPK, TGF-ß or PI3K signaling. Ano1 can indirectly be targeted via inhibition of the intra-cellular domain of EGFR with afatinib. Scheme created with BioRender.

## Supplementary Tables

**Supplementary Table 1:** D0 and Area Under the Curve (AUC) for each cell line, as well as their specific genotypes.

| Cell Lines | Genotype | D0 | AUC |
|---|---|---|---|
| **FaDu_c5** | *CCND1* / *ANO1* amplified | 2.94 ± 0.05 | 2.59 |
| **FaDu_c46** | *CCND1* / *ANO1* amplified | 2.11 ± 0.29 | 2.01 |
| **FaDu_c54** | *CCND1* / *ANO1* amplified | 1.86 ± 0.05 | 1.88 |
| **FaDu_c78** | *CCND1* / *ANO1* amplified | 2.83 ± 0.22 | 2.51 |
| **UM-SCC-22B** | *CCND1* / *ANO1* amplified | 1.47 ± 0.22 | 1.61 |
| **c5-LeGo** | *CCND1* / *ANO1* amplified | 2.70 ± 0.13 | 2.4 |
| **c5-KD *CCND1*** | *CCND1* non-amplified / *ANO1* amplified | 2.55 ± 0.15 | 2.29 |
| **c5-KD *ANO1*** | *CCND1* amplified / *ANO1* non-amplified | 2.00 ± 0.23 | 1.96 |

**Supplementary Table 2:** TCGA HNSCC subgroup analysis based on Cyclin D1 and Ano1 expression reveals differences in expression levels of genes regulating DNA damage repair: BRCA1, RAD51B and BRCA2 are involved in homologous recombination (HR), whereas XRCC6 is part of the non-homologous end-joining (NHEJ).

| Gene | Cytoband | Log2 Ratio (high vs low expression group) | p-Value | q-Value | Higher expression in |
|---|---|---|---|---|---|
| **BRCA1** | 17q21.31 | 0.28 | 1.67E-04 | 2.56E-03 | $Ano1^{high}$ |
| **RAD51B** | 14q24.1 | 0.21 | 3.50E-04 | 4.30E-03 | $Ano1^{high}$ |
| **BRCA2** | 13q13.1 | 0.42 | 8.56E-04 | 8.36E-03 | $Ano1^{high}$ |
| **XRCC6** | 22q13.2 | 0.15 | 1.95E-03 | 0.0411 | Cyclin $D1^{high}$ |

## Supplementary Materials and Methods.

### Media composition.

| Cell lines | Media |
|---|---|
| FaDu<br>UM-SCC-22B<br>UT-SCC-9<br>UT-SCC-15<br>(HNSCC) | Minimum Essential Media (MEM, Thermo Fisher Scientific)<br>12% (vol/vol) Fetal Bovine Serum (FBS, Thermo Fisher Scientific)<br>1x Non-Essential Amino Acids Solution (NEAA, Thermo Fisher Scientific) |
| HEK293T | Advanced DMEM/F12 (Thermo Fisher Scientific)<br>10% FBS<br>1% Penicillin/Streptomycin (Merck)<br>1% L-Glutamine (Thermo Fisher Scientific)<br>1 mM Sodium Pyruvate (Thermo Fisher Scientific)<br>20 mM HEPES (Merck) |

**Droplet Digital PCR.** The ddPCR was used for the duplex assay composed of two sets of primers (900 nM), probes (250 nM) and 10 ng of sample DNA. A threshold was set at >10,000 droplets when analyzing the experiments.

| Reagents / Material | Reference |
|---|---|
| DNeasy Blood & Tissue Kit | Qiagen |
| ddPCR™ Supermix for Probes no dUTP | BioRad |
| Droplet Generator | BioRad QX200™ |
| Droplet Reader | BioRad QX200™ |

| Steps | Temperature [°C] | Time | Number of cycles |
|---|---|---|---|
| Initial denaturation | 95 | 10 min | 1 |
| Denaturation | 94 | 30 sec | 40 |
| Annealing-Elongation | 60 | 1 min | |
| Enzyme deactivation | 98 | 10 min | 1 |
| Hold | 4 | Minimum 10 min | 1 |

| Genes | Primer Forward (5’→3‘) | Primer Reverse (5’→3‘) |
|---|---|---|
| *HBB* | CCTGTGGAGCCACACCCTA | GATGGCTCTGCCCTGACTTT |
| *CCND1* | GAGGATGTTCATAAGGCCAGTA | CTGTAACATCAAAGGCAGAAGG |
| *ANO1* | GAATGACTGAGAGTGTAGGGC | GACTTCCATTCTGCCAAGGTC |

| Genes | Probe (5’→3‘) |
|---|---|
| *HBB* | 6FAM_CTCCCAGGAGCAGGGAGGGC_BHQ1 |
| *CCND1* | HEX_ACACACACACACACAC_BHQ1 |
| *ANO1* | HEX_CTCAGAGGCATTGGGAAGCTTGCTGT_BHQ1 |

**RT-qPCR.** 500 ng of RNA was used for complementary DNA (cDNA) conversion. The cDNA template was mixed with 200 nM of forward and reverse primers exon-exon junction spanning and 200 nM of probes. The dual fluorescence (HEX for gene of interest and FAM for reference gene) was acquired during the elongation step. Cp values were used for $2^{-\Delta\Delta Cq}$ (Livak) method expression level calculation.

| Reagents / Material | Reference |
|---|---|
| High Pure RNA Isolation Kit | Roche |
| Omniscript RT Kit | Qiagen |
| LightCycler 480 Probes Master (2x conc.) | Roche |
| LightCycler 480 II system | Roche |

| Steps | Temperature [°C] | Time | Number of cycles |
|---|---|---|---|
| Initial denaturation | 95 | 10 min | 1 |
| Denaturation | 95 | 10 sec | 45 |
| Annealing | 60 | 30 sec | |
| Elongation | 72 | 1 sec | |
| Hold | 4 | 10 sec | 1 |

| Genes | Primer Forward (5'→3') | Primer Reverse (5'→3') |
|---|---|---|
| *GAPDH* | GCACCACCAACTGCTTAGCA | GTCTTCTGGGTGGCAGTGATG |
| *SDHA* | CGAGGTTTTCACTTCACTGTT | ACCACCACTGCATCAAATTC |
| *PBGD* | TGCCCTGGAGAAGAATGAAG | CAGCATCATGAGGGTTTTCC |
| *CCND1* | GGATGCTGGAGGTCTGCGA | AGAGGCCACGAACATGCAAG |
| *ANO1* | GAGCCAAAGACATCGGAATCTG | TGAAGGAGATCACGAAGGCAT |

| Genes | Probe (5'→3') |
|---|---|
| *GAPDH* | FAM_TCGTGGAAGGACTCATGACCACAGTCC_BHQ1 |
| *SDHA* | FAM_ATTCCATTTCTGCTCAGTATCCAGTAGTGG_BHQ1 |
| *PBGD* | FAM-AAGGACCTGCCCACTGTGCTTCC--BHQ1 |
| *CCND1* | HEX_AGGAGGTCTTCCCGCTGGCCATGAAC_BHQ1 |
| *ANO1* | HEX_CTCAGAGGCATTGGGAAGCTTGCTGT_BHQ1 |

**Western Blot.** Proteins were extracted from the cells with RIPA buffer complemented with 1% of Halt™ Protease and Phosphatase Inhibitor Cocktail 100X (Thermo Fisher Scientific) and measured with Pierce BCA Protein Assay (Thermo Fisher Scientific). 30 µg of proteins were loaded on an 8% SDS-PAGE gel and transferred on a 0.45 µm PVDF membrane (Merck Millipore, Burlington, MA, USA) with a semi-dry transfer (Trans-blot, BioRad). After blocking, the membranes were incubated over-night at 4°C with primary antibody. The next day, secondary antibody was incubated and the signal was detected by adding chemiluminescent substrate (WesternBright Sirius HRP, Advansta, San Jose, CA, USA) and imaged with ChemiDoc™ MP Imaging System (BioRad). Lines were quantified with Image Lab 6.0 (BioRad).

| Protein | Dilution | Reference |
|---|---|---|
| Cyclin D1 | 1:500 | Abcam [SP4] ab16663 (Cambridge, UK) |
| Vinculin | 1:1000 | Abcam [EPR8185] |
| α-tubulin | 1:1000 | Abcam [EPR13478(B)] |
| Phospho-retinoblastoma Ser780 | 1:1000 | Cell Signaling Technology [C84F6] (Danvers, MA, USA) |
| Phospho-Cyclin D1 Thr286 | 1:1500 | Cell Signaling Technology [D29B3] |
| Peroxidase Conjugated AffiniPure Goat anti-rabbit IgG | 1:15,000 | Jackson ImmunoResearch Labs (West Grove, PA, USA) |

**shRNA sequences.** Five shRNA targeting the genes of interest (see sequences below) were bought as bacterial stock from MISSION® shRNA Bacterial Glycerol Stock (Merck) in pLKO.1-puro (7,1 kbp) or TRC2-pLKO-puro (7,5 kbp) vectors containing a puromycin resistance gene. The shRNA present in the pLKO were used for cloning into the LeGo vector. Control vector was the LeGo-GFP-Puro backbone cloned with a scramble RNA sequence (SHC002), designed to not target any known gene in the cell. To insure specificity of the targeting shRNA, sequences were aligned with NCBI-BLAST which confirmed absence of homology sequence to other gene. To validate the shRNA construct, specificity score was calculated from the TRC database (Broad Institute: https://portals.broadinstitute.org/gpp/public/) which confirmed absence of off-target matches. The ligated plasmids were then transformed into competent *E. Coli* (MAX Efficiency DH5α Competent cells, Thermo Fisher Scientific) and purified (QIAprep Miniprep, Qiagen).

| | |
|---|---|
| ***CCND1*** | shRNA #1: TGTTGTAAGAATAGGCATTAACTCGAGTTAATGCCTATTCTTACAACAT<br>shRNA #2: CCACAGATGTGAAGTTCATTTCTCGAGAAATGAACTTCACATCTGTGGT<br>shRNA #3: TCCTCTCCAGAGTGATCAAGTCTCGAGACTTGATCACTCTGGAGAGGAT<br>shRNA #4: ACAACTTCCTGTCCTACTACCCTCGAGGGTAGTAGGACAGGAAGTTGTT<br>shRNA #5: GAACAAACAGATCATCCGCAACTCGAGTTGCGGATGATCTGTTTGTTCT |
| ***ANO1*** | shRNA #1: GGTGGCTGGTAATACGGCAATAACTCGAGTTATTGCCGTATTACCAGCCATT<br>shRNA #2: TGCAGATCTGCAGGTATAAAGCTCGAGCTTTATACCTGCAGATCTGCATT<br>shRNA #3: CTGATGCCGAGTGCAAGTATGCTCGAGCATACTTGCACTCGGCATCAGTT<br>shRNA #4: GGCGTCGAGTTCAACGACAGAAACTCGAGTTTCTGTCGTTGAACTCGACGTT<br>shRNA #5: GGCATCGGAATCTGGTACAATATCTCGAGATATTGTACCAGATTCCGATGTT |
| **SHC002** (scramble RNA) | TAAGGTTAAGTCGCCCTCGCTCGAGCGAGGGCGACTTAACCTT |

**Transfection of lentivirus.** HEK293T cells were cotransfected with 40 µg.mL$^{-1}$ of polyethylenimine PEI (Thermo Fisher Scientific), 1 µg of plasmid (LeGo-GFP-Puro-shRNA) and 1 µg of packaging plasmids at a 1:1:1 ratio of: pRSV-Rev (Addgene, Watertown, MA, USA), pCMV-VSV-G (Addgene), and pMDLg/pRRE (Addgene). The supernatant containing the packaged lentivirus was collected at 48 and 72 hours, purified with a 0.45 µm filter to remove cells and other debris and snap-frozen in liquid nitrogen.

**Cell transduction and generation of stable cell lines.** The packaged lentivirus was then transduced on HNSCC cell lines in the presence of 8 µg.ml polybrene (Merck) followed by a spinoculation step (1000 x g, 1 hour, 25°C). The viral titration method was used to calculate the MOI (Multiplicity of Infection) and was established by transducing HEK293T cells and analyzing the amount of GFP positive-cells after 48 hours via flow cytometry. After 72 hours post transduction, puromycin (Roth) selection started. The right puromycin concentration was chosen based on a kill curve analysis previously made on the same cell line used for transduction. 24 hours later, a limiting dilution was carried out to isolate a monoclonal cell population. Gene expression analysis was done regularly to monitor the stability and efficiency of the knockdown.

## Supplementary Images

**Supplementary Image S1:** Original blot from Figure 6a.

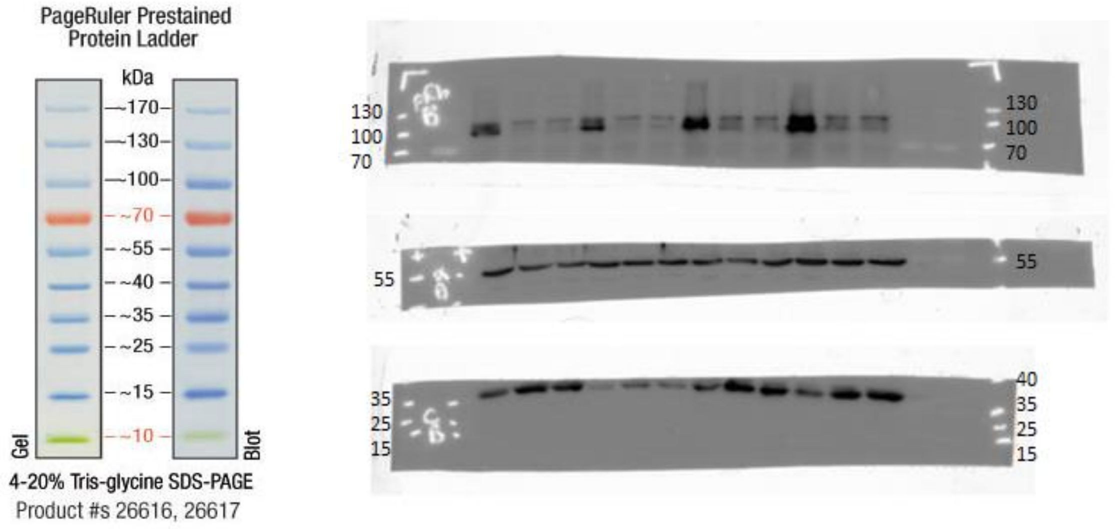


**Supplementary Image S2:** Original blot from Figure 7a.

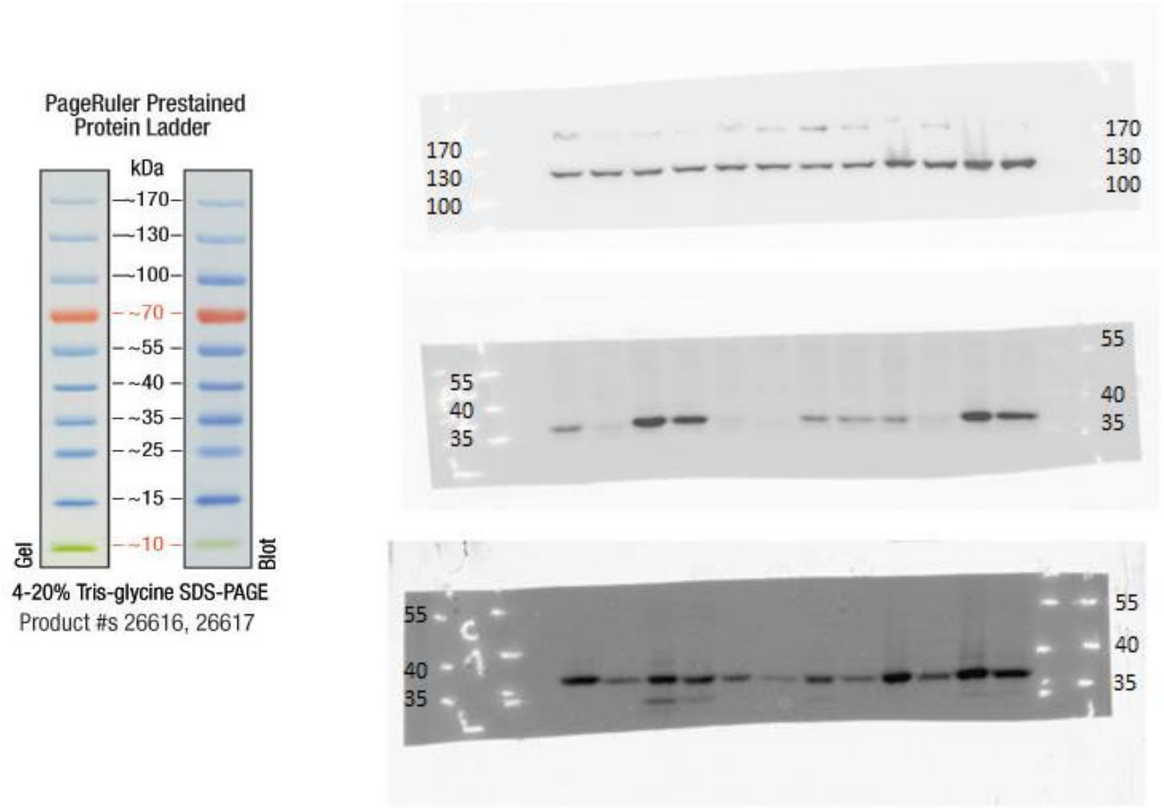

Supplement: Supplementary file 1 — Supplementary Material 1 [file 41598_2025_85214_MOESM1_ESM.pdf]
